# Deep Learning Approaches to Surrogates for Solving the Diffusion Equation for Mechanistic Real-World Simulations

**DOI:** 10.3389/fphys.2021.667828

**Published:** 2021-06-24

**Authors:** J. Quetzalcóatl Toledo-Marín, Geoffrey Fox, James P. Sluka, James A. Glazier

**Affiliations:** ^1^Biocomplexity Institute, Indiana University, Bloomington, IN, United States; ^2^Luddy School of Informatics, Computing and Engineering, Bloomington, IN, United States; ^3^Digital Science Center, Bloomington, IN, United States

**Keywords:** diffusion surrogate, machine learning, virtual tissue, mechanistic modeling, Julia

## Abstract

In many mechanistic medical, biological, physical, and engineered spatiotemporal dynamic models the numerical solution of partial differential equations (PDEs), especially for diffusion, fluid flow and mechanical relaxation, can make simulations impractically slow. Biological models of tissues and organs often require the simultaneous calculation of the spatial variation of concentration of dozens of diffusing chemical species. One clinical example where rapid calculation of a diffusing field is of use is the estimation of oxygen gradients in the retina, based on imaging of the retinal vasculature, to guide surgical interventions in diabetic retinopathy. Furthermore, the ability to predict blood perfusion and oxygenation may one day guide clinical interventions in diverse settings, i.e., from stent placement in treating heart disease to BOLD fMRI interpretation in evaluating cognitive function (Xie et al., 2019; Lee et al., 2020). Since the quasi-steady-state solutions required for fast-diffusing chemical species like oxygen are particularly computationally costly, we consider the use of a neural network to provide an approximate solution to the steady-state diffusion equation. Machine learning surrogates, neural networks trained to provide approximate solutions to such complicated numerical problems, can often provide speed-ups of several orders of magnitude compared to direct calculation. Surrogates of PDEs could enable use of larger and more detailed models than are possible with direct calculation and can make including such simulations in real-time or near-real time workflows practical. Creating a surrogate requires running the direct calculation tens of thousands of times to generate training data and then training the neural network, both of which are computationally expensive. Often the practical applications of such models require thousands to millions of replica simulations, for example for parameter identification and uncertainty quantification, each of which gains speed from surrogate use and rapidly recovers the up-front costs of surrogate generation. We use a Convolutional Neural Network to approximate the stationary solution to the diffusion equation in the case of two equal-diameter, circular, constant-value sources located at random positions in a two-dimensional square domain with absorbing boundary conditions. Such a configuration caricatures the chemical concentration field of a fast-diffusing species like oxygen in a tissue with two parallel blood vessels in a cross section perpendicular to the two blood vessels. To improve convergence during training, we apply a training approach that uses roll-back to reject stochastic changes to the network that increase the loss function. The trained neural network approximation is about 1000 times faster than the direct calculation for individual replicas. Because different applications will have different criteria for acceptable approximation accuracy, we discuss a variety of loss functions and accuracy estimators that can help select the best network for a particular application. We briefly discuss some of the issues we encountered with overfitting, mismapping of the field values and the geometrical conditions that lead to large absolute and relative errors in the approximate solution.

## 1. Introduction

Diffusion is ubiquitous in physical, biological, and engineered systems. In mechanistic computer simulations of the dynamics of such systems, solving the steady state and time-varying diffusion equations with multiple sources and sinks is often the most computationally expensive part of the calculation, especially in cases with multiple diffusing species with diffusion constants differing by multiple orders of magnitude. Examples in biology include cells secreting and responding to diffusible chemical signals during embryonic development, blood vessels secreting oxygen which cells in tissues absorb during normal tissue function, tumors secreting growth factors promoting neoangiogenesis in cancer progression, or viruses spreading from their host cells to infect other cells in tissues. In these situations the natural diffusion constants can range from ~ 10^3^μm^2^/s for oxygen to ~ 0.1−10^2^μm^2^/s for a typical protein (Phillips, 2018). Dynamic simulations of biological tissues and organs may require the independent calculation of the time-varying concentrations of dozens of chemical species in three dimensions, and in the presence of a complex field of cells and extracellular matrix. As the number of species increases, solving these diffusion equations dominates the computational cost of the simulation. Numerous approaches attempt to reduce the cost of solving the diffusion equation including implicit, particle-based, frequency-domain and finite-element methods, multithreaded, and MPI-based parallelization and GPUs, but all have significant limitations. HPC methods that do not require Deep Learning (DL) can certainly accelerate solution of problems including diffusion equations, e.g., Secomb's Green's function method leverages GPUs to accelerate solution of 3D advection-diffusion in microvessels with time-dependent sinks and sources (Secomb, 2016). Such methods could greatly reduce the time required to generate training sets for DL-assisted approaches. Machine learning has also been applied to solve a growing list of PDE problems (Farimani et al., 2017; Sharma et al., 2018; Edalatifar et al., 2020; He and Pathak, 2020; Li A. et al., 2020; Li Z. et al., 2020; Cai et al., 2021). See Fox and Jha (2019) for a thorough review. Machine learning has also been applied to the *inverse problem*, i.e., attempting to infer the underlying mechanistic equations governing a complex system from experimental data. These methods can potentially lead to the discovery of new physics (Champion et al., 2019). Similarly, Neural-ODEs is a highly active and exciting field where neural networks are embedded into a differential equation. Modeling a process via an ODE typically consists in equating the change rate of a *quantity* (e.g., concentration) to an operator applied to that same *quantity* plus some other *quantities* which are called inhomogeneities or *external fields*, then solving the ODE and comparing it with experimental data of that *thing* to validate the model or fit parameters. The operator in the ODE is selected *a priori* based on the *symmetries* of the process. Neural-ODEs replaces the operator with a neural network. The neural network is trained by solving the Neural-ODE and comparing it with the experimental data (Chen et al., 2018; Rackauckas et al., 2019). Moreover, *Physics Informed Neural Networks* tackle forward and inverse problems by embedding physical information into the neural network. Embedding physical information into the neural network means embedding the ODE, the initial conditions and the boundary conditions into the loss function used to train the neural network (Raissi et al., 2019). In the case of multiscale modeling, the complexity of the system includes different characteristic length and time scales differing by orders of magnitude. Multiscale modeling using standard computational approaches, such as Monte Carlo methods and/or molecular dynamics is time consuming. AI-based surrogates using deep learning methods can accelerate computation by replacing specific classical solvers, while preserving the overall interpretability of mechanistic models. In real-world problems, the number of sources and sinks, their shape, boundary fluxes, and positions differ from instance to instance and may change in time. Boundary conditions may also be complicated and diffusion constants may be anisotropic or vary in space. The resulting lack of symmetry means that many high-speed implicit and frequency-domain diffusion-solver approaches do not work effectively, requiring the use of simpler but slower forward solvers (Schiesser, 2012). Deep learning^1^ surrogates to solve either the steady-state field or the time-dependent field for a given set of sources and sinks subject to diffusion could potentially increase the speed of such simulations by several orders of magnitude compared to the use of direct numerical solvers.

**Figure 1:**
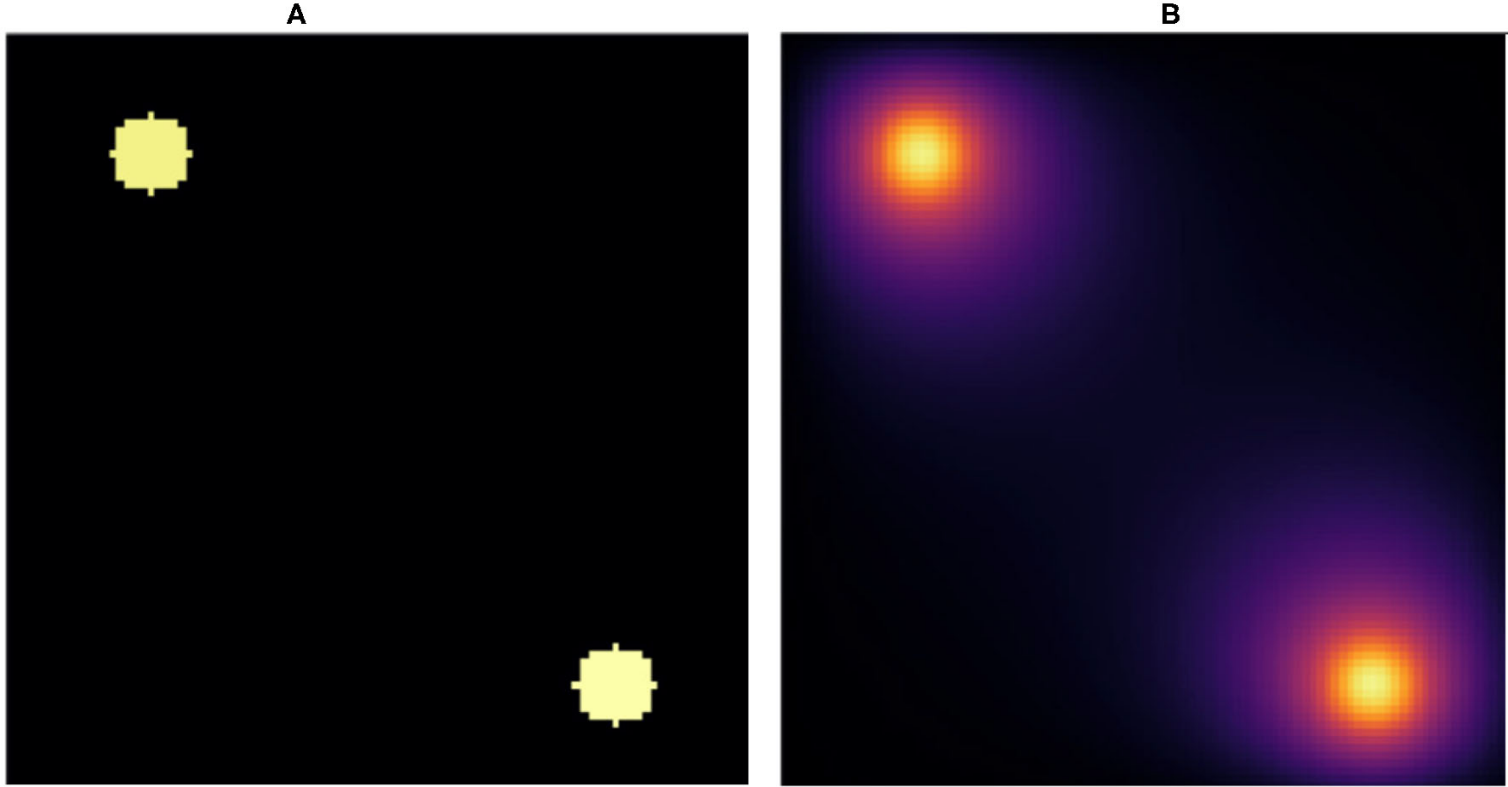
Snapshot of **(A)** initial condition and **(B)** stationary state solution. **(A)** We placed two random value sources of radius 5*voxels* in random positions fully within a 100 × 100*pixel* lattice and used this configuration as the input to the NN. **(B)** Stationary solution to the diffusion equation with absorbing boundary conditions for the initial conditions in **(A)**. The stationary solution **(B)** is the target for the NN. We fixed the diffusion constant to *D* = 1*voxels*^2^/*s* and the decay rate to γ = 1/400*s*^−1^, which yields a diffusion length equal to $\sqrt{D/\gamma }voxels=20voxels$.

One challenge in developing effective deep neural network (NN) diffusion-solver surrogates is that the dimensionality of the problem specification is potentially very high, with an arbitrary pattern of sources and sinks, with different boundary conditions for each source and sink, and spatially variable or anisotropic diffusivities. As a proof-of-principle we will start with a NN surrogate for a simple version of the problem that we can gradually generalize to a full surrogate in future work. In a two-dimensional square domain represented as *N* × *Npixels* and with absorbing boundary conditions, we place two circular sources of equal diameters at random positions, with the constraint that the sources do not overlap and are fully contained within the domain. Each source imposes a constant value on the diffusing field within the source and at its boundary. We select the value for one of the sources equal to 1 while the value for the other source is randomly selected from a uniform distribution between (0, 1] (see Figure 1A). Outside the sources the field diffuses with a constant diffusion constant (*D*) and linearly decays with a constant decay rate (γ). This simple geometry could represent the diffusion and uptake of oxygen in a volume of tissue between two parallel blood vessels of different diameters. Although reflecting or periodic boundary conditions might better represent a potion of a larger tissue, we use the simpler absorbing boundary conditions here. In this case, the steady-state field depends critically on the distance between the sources, and between the sources and the boundary, both relative to the diffusion length ($l_{D}={\left(D/\gamma \right)}^{1/2}$) and on the sources' field strengths.

In practice then, the solution of the steady state diffusion equation maps an image consisting of *N* × *N* pixels with 0 value outside the sources and constant values between 0 and 1 inside the sources to a second image of the same size, which has the same values inside the sources but values between 0 and 1 elsewhere (see Figure 1B). We evaluate the ability of a NN trained on the explicit numerical solutions of the steady-state diffusion field for 20, 000 two-source examples to approximate the steady state field for configurations of sources that it had not previously encountered.

Notice that the diffusion kernel convolution used in the direct solution of the time-dependent diffusion equation (e.g., finite-element methods) is a type of convolutional neural network (Schiesser, 2012). Therefore we chose deep convolutional NN as the architecture. However, there are multiple types of convolutional NN. Here we considered two of these. A deep convolutional neural network and an autoencoder (Baur et al., 2020). In addition, because it was possible that these two types would do better at replicating specific aspects of the overall solution, we also evaluated a superposition of the two. Time series surrogates often use recurrent NN (Zhang and Xiao, 2000; Dubois et al., 2020). Similarly, deep generative models have been shown to be useful to sample from high dimensional space, as in the case of molecular dynamics and chemical reaction modeling (Chen and Ferguson, 2018; Noé et al., 2019, 2020; Zhang et al., 2019; Gkeka et al., 2020; Kasim et al., 2020). Since our main interest is the stationary solution, we did not consider these approaches.

## 2. Model

**Figure 2:**
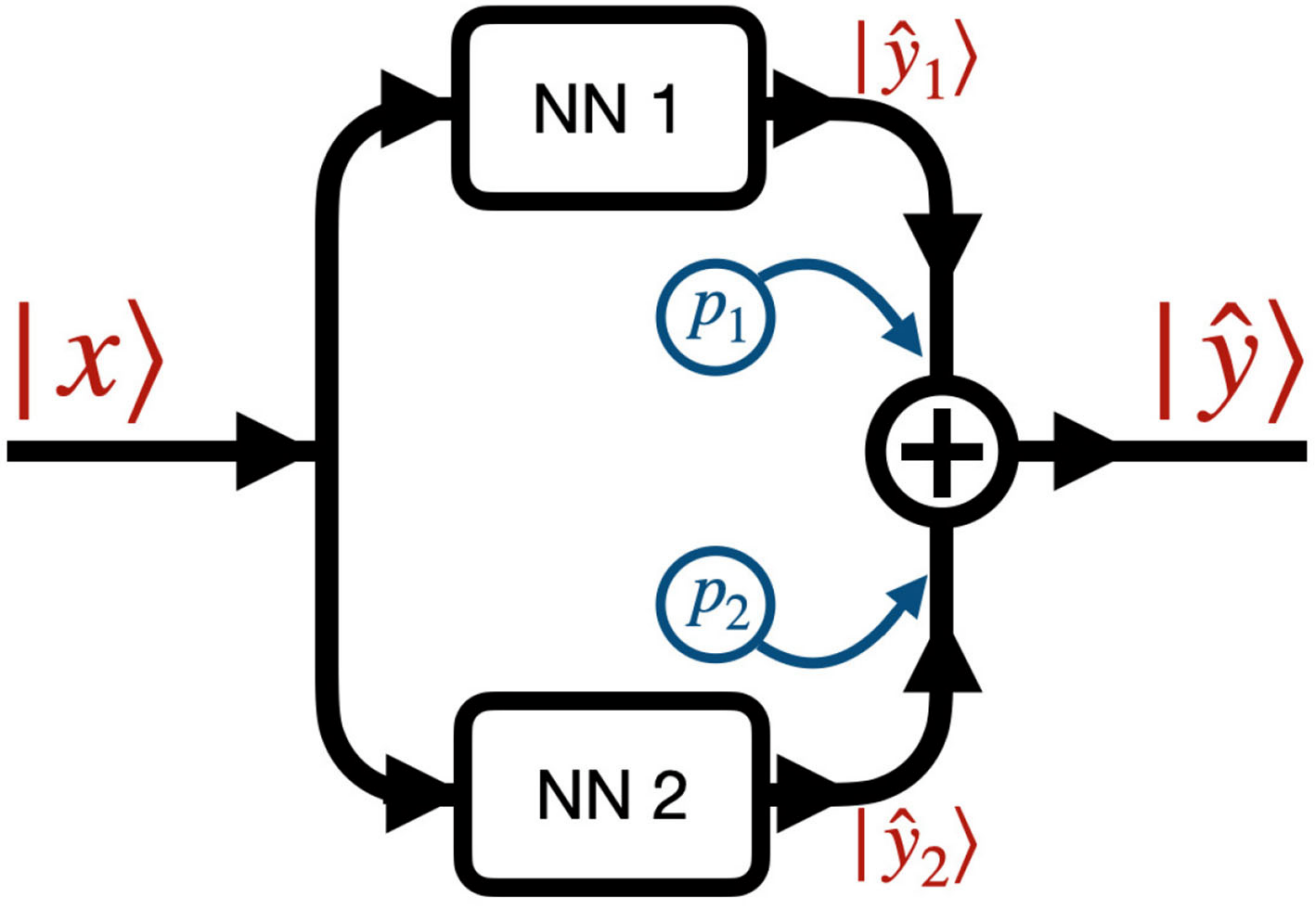
Network architecture: the input image |*x*〉 passes through NN 1 (see Figure 3A) and NN 2 (see Figure 3B), generating the two outputs ŷ_1_〉 and |ŷ_2_〉. The final output |ŷ〉 is the sum of the outputs of the two NNs weighted by coefficients *p*_1_ and *p*_2_, i.e., |ŷ〉 = *p*_1_|ŷ_1_〉+*p*_2_|ŷ_2_〉. *p*_*i*_ are fixed Boolean hyperparameters for the model and fixed for each model we trained. This means that when a given model has *p*_*i*_ = 0 (*p*_*i*_ = 1) then *NNi* is turned off (on).

**Figure 3:**
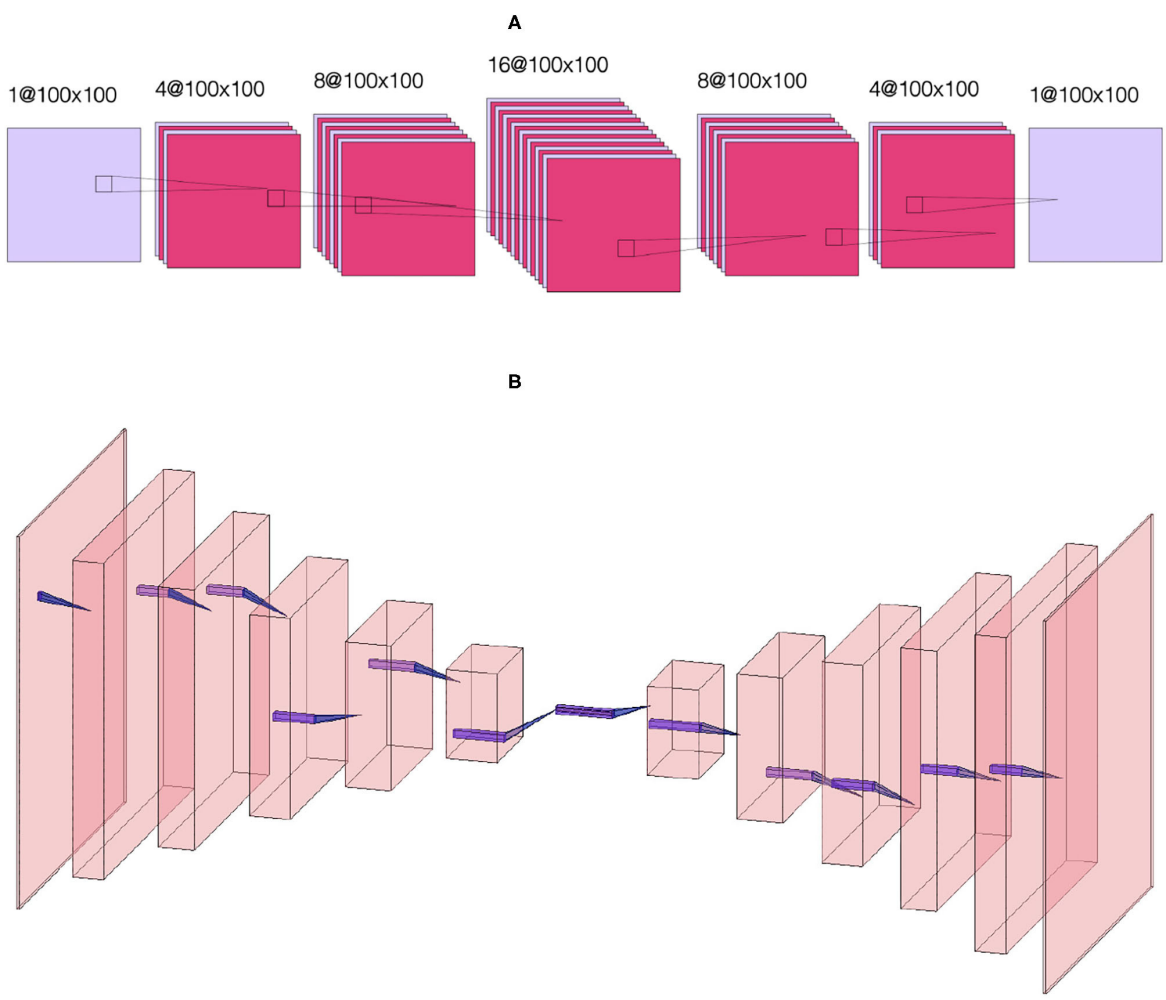
Sketch of **(A)** convolutional NN 1. The first layer takes as input a single-channel *N* × *N* image and applies four 3 × 3 convolutions to generate four *N* × *N* images, the second layer applies eight 3 × 3 convolutions to generate eight *N* × *N* images, the third layer applies 16 3 × 3 convolutions to generate sixteen *N* × *N* images, the fourth layer applies eight 3 × 3 convolutions to generate eight *N* × *N* images, the fifth layer applies four 3 × 3 convolutions to generate four *N* × *N* images and the sixth layer applies a 3 × 3 convolution to generate a single *N* × *N* image. Sketch of **(B)** autoencoder NN 2. The first six layers perform a meanpool operation that reduces image height and width by half after each layer, with the image dimensions following the sequence {100^2^, 50^2^, 25^2^, 12^2^, 6^2^, 3^2^, 1^2^} while adding channels after each layer following the sequence {1, 64, 128, 256, 512, 1024, 2048}. Then, the following six layers reverse the process, reducing the number of channels following the sequence {1024, 512, 256, 128, 64, 1} while increasing the height and width following the sequence {1^2^, 3^2^, 7^2^, 13^2^, 25^2^, 51^2^, 100^2^}. This sketch only defines the kinds of layers used. For details about the activation functions used in each layer (see Table 1).

Figure 2 shows the data flow through the NN. We denote by |*x*〉 and |ŷ〉 the input and output images, that is the initial condition layout of the source cells and the predicted stationary solution of the diffusion equation, respectively. The input |*x*〉 passes to two different neural networks (NNs) denoted *NN 1* (Figure 3A) and *NN 2* (Figure 3B) which output |ŷ_1_〉 and |ŷ_2_〉, respectively. The output |ŷ〉 is a weighted sum of the outputs of the two NNs, |ŷ〉 = *p*_1_|ŷ_1_〉+*p*_2_|ŷ_2_〉, where *p*_1_ and *p*_2_ are fixed hyperparameters, i.e., these hyperparameters are fixed during training. In our code (Toledo-Marin, 2020) *p*_*i*_ are real numbers, however, in this paper we only consider the Boolean case where they each take values of 0 or 1. *NN 1* is a deep convolutional neural network that maintains the height and width of the input image through each of 6 convolutional layers. The first layer outputs a 4-channel image, the second layer outputs an 8-channel image, the third layer outputs a 16-channel image, the fourth layer outputs an 8-channel image, the fifth layer outputs a 4-channel image and the sixth layer outputs a 1-channel image. *NN 2* is an autoencoder (Chen et al., 2017) where the first six layers perform a meanpool operation that reduces height and width in half after each layer following the sequence {100^2^, 50^2^, 25^2^, 12^2^, 6^2^, 3^2^, 1^2^} while adding channels after each layer following the sequence {1, 64, 128, 256, 512, 1, 024, 2, 048}. Then, the following six layers consist on reducing the number of channels following the sequence {1, 024, 512, 256, 128, 64, 1} while increasing the height and width following the sequence {1^2^, 3^2^, 7^2^, 13^2^, 25^2^, 51^2^, 100^2^}. Figure 3 sketches the architectures of the two NNs, while Table 1 provides their parameters. We will find that NN 1 will capture the sources whereas NN 2 will capture the field. In Table 1, we specify each neural network by specifying for each layer the kind of layer, the activation function and the output shape.

**Table 1:** Convolutional neural network architectures.

**Operation**	**Act**	**Output shape**
Conv 3 × 3	LReLU	4 × 100 × 100
Dropout 1 (*D*_1_)	–	–
BatchNorm	Identity	–
Conv 3 × 3	LReLU	8 × 100 × 100
BatchNorm	Identity	–
Conv 3 × 3	LReLU	16 × 100 × 100
BatchNorm	Identity	–
Conv 3 × 3	LReLU	8 × 100 × 100
BatchNorm	Identity	–
Conv 3 × 3	LReLU	4 × 100 × 100
BatchNorm	Identity	–
Conv 3 × 3	ReLU	1 × 100 × 100
Dropout 2 (*D*_2_)	–	–
BatchNorm	Identity	–
Conv 3 × 3	LReLU	64 × 100 × 100
BatchNorm	Identity	–
Dropout 3 (*D*_3_)	–	–
Meanpool	Identity	64 × 50 × 50
Conv 3 × 3	LReLU	128 × 50 × 50
Meanpool	Identity	128 × 25 × 25
Conv 3 × 3	LReLU	256 × 25 × 25
Meanpool	Identity	256 × 12 × 12
Conv 3 × 3	LReLU	512 × 12 × 12
Meanpool	Identity	512 × 6 × 6
Conv 3 × 3	LReLU	1,024 × 6 × 6
Meanpool	Identity	1,024 × 3 × 3
Conv 3 × 3	LReLU	2,048 × 1 × 1
ConvT 3 × 3	LReLU	1,024 × 3 × 3
ConvT 3 × 3	LReLU	512 × 7 × 7
ConvT 3 × 3	LReLU	256 × 13 × 13
ConvT 3 × 3	LReLU	128 × 25 × 25
ConvT 3 × 3	LReLU	64 × 51 × 51
Dropout 4 (*D*_4_)	–	–
ConvT 4 × 4	ReLU	1 × 100 × 100
BatchNorm	Identity	–

*Left panel corresponds to the successive operations of NN 1 while the right panel corresponds to the successive operations NN 2. Act stands for activation function. Conv, ConvT, and (L)ReLU stand for convolution, convolution transpose, and (leaky) rectified linear unit, while Identity means the activation function is the identity function (see Innes et al., 2018). Both NNs take as input the initial condition which has dimensions Channels × Width × Height = 1 × 100 × 100*.

To generate representative two-source initial conditions and paired steady-state diffusion fields, we considered a two-dimensional lattice of size 100 × 100*units*^2^. We generated 20 k configurations with two sources, each with a radius of 5*units*. One source has a constant source value equal to 1, while the other source has a constant source value between 0 and 1 randomly assigned using a uniform distribution. Everywhere else the field value is 0. We placed the sources in randomly uniform positions in the lattice. This image served as the input for the NN |*x*〉. Then we calculated the stationary solution to the diffusion equation with absorbing boundary conditions for each initial condition using the *Differential Equation* package in Julia (Rackauckas and Nie, 2017). The Julia-calculated stationary solution is the target or ground truth image for the NN |*y*〉. In Figures 1A,B, we show an initial condition and the stationary solution, respectively. We have set the diffusion constant to *D* = 1*units*^2^/*s* and the decay rate γ = 1/400*s*^−1^, which yield a diffusion length $l_{D}=\sqrt{D/\gamma }=20units$. Notice that this length is 4 times the radius of the sources and 1/5 the lattice linear dimension. As γ increases and as *D* decreases, this length decreases. As this length decreases, the field gradient also decreases (Tikhonov and Samarskii, 2013). The source code to generate the data and train the NN can be found in Toledo-Marin (2020).

**Figure 4:**
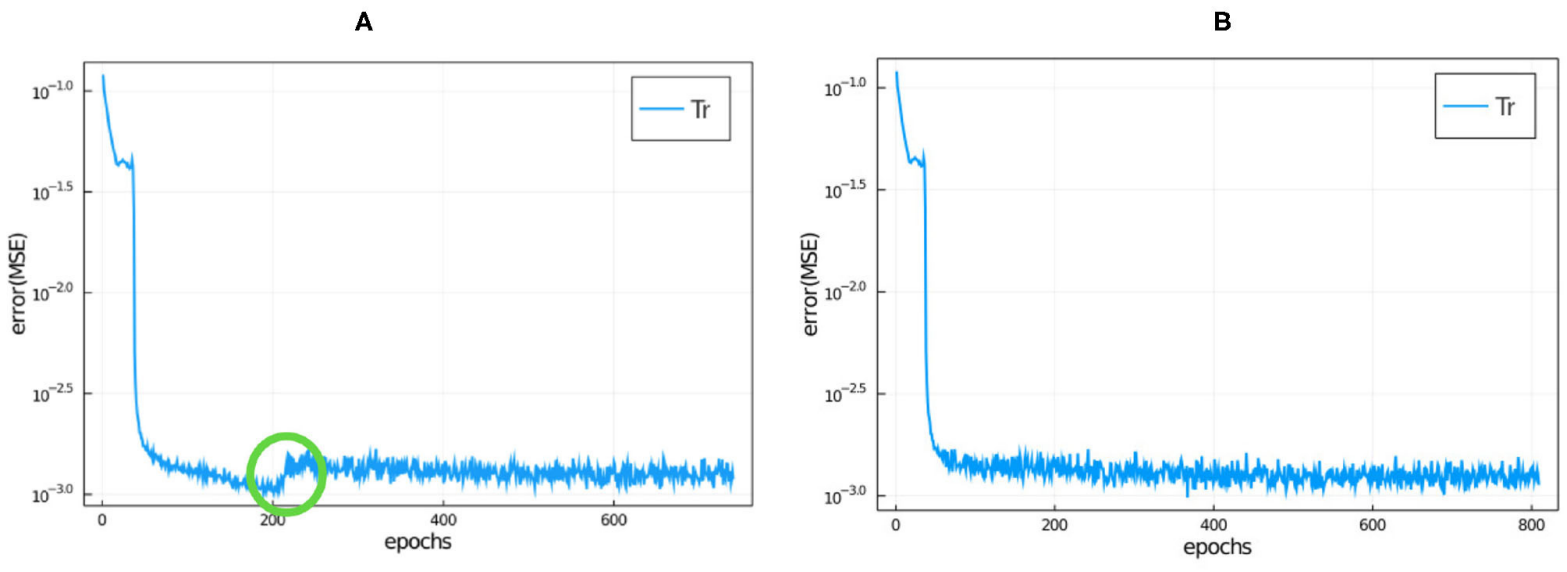
Training loss function vs. epochs for model 9 (the hyperparameters are specified in Table 2 and the NN details are described in the main text) without roll-back **(A)** and with roll-back **(B)** using the same seed. We have circled in green where a jump occurred during this training run (see main text for discussion).

We trained the CNN setting the number of epochs to 800 using the deep learning library in Julia called Flux (Innes, 2018). We varied the dropout values between 0.0 and 0.6 in steps of 0.1 (see Table 2). We used ADAM as the optimizer (Kingma and Ba, 2014). Deciding on a loss function is a critical choice in the creation of the surrogate. The loss function determines the types of error the surrogate's approximation will make compared to the direct calculation and the acceptability of these errors will depend on the specific application. The mean squared error (*MSE*) error is a standard choice. However, it is more sensitive to larger absolute errors and therefore tolerates large relative errors at pixels with small values. A loss function calculated on the log of the values would be equally sensitive to relative error no matter what the absolute value. In most biological contexts we want to have a small absolute error for small values and a small relative error for large values. We explored the use of both functions, *MAE* and *MSE*, as described in Table 2. We used 80 and 20% of the dataset for training and test sets, respectively. We trained each model once. The highest and lowest values in the input and output images are 1 and 0, respectively. The former only occurs in sources and their vicinity. Given the configurations of the sources, the fraction of pixels in the image with values near 1 is ~ 2π*R*^2^/*L*^2^ ≈ 2%. Thus, pixels with small values are much more common than pixels with large values, and because the loss function is an average over the field, high field values tend to get washed out. To account for this unbalance between the frequency of occurrence of low and high values, we introduced an exponential weight on the pixels in the loss function. We modulate this exponential weight through a scalar hyperparameter *w*, for the field in the *i*th lattice position in the loss function as

(1) $$\begin{array}{r} L_{i\beta }^{\left(\alpha \right)}=\exp\left(-\left(\langle i|1\rangle -\langle i|y_{\beta }\rangle \right)/w\right)\cdot {\left(\langle i|{ŷ}_{\beta }\rangle -\langle i|y_{\beta }\rangle \right)}^{\alpha }, \end{array}$$

where α is 1 or 2 for MAE or MSE, respectively and β tags the tuple in the data set (input and target). Here 〈|〉 denotes the inner product and |*i*〉 is a unitary vector with the same size as |*y*_β_〉 with all components equal to zero except the element in position *i* which is equal to one. |1〉 is a vector with all components equal to 1 and with size equal to that of |*y*_β_〉. Then 〈*i*|*y*_β_〉 is a scalar corresponding to the pixel value at the *i*th position in |*y*_β_〉, whereas 〈*i*|1〉 = 1 for all *i*. Notice that high pixel values will then have an exponential weight ≈1 while low pixel values will have an exponential weight ≈exp(−1/*w*). This implies that the error associated to high pixels will have a larger value than that for low pixels. The loss function $L^{\left(\alpha \right)}$ is the mean value over all pixels (*i*) and a given data set (β):

(2) $$\begin{array}{r} L^{\left(\alpha \right)}=\langle L_{i\beta }^{\left(\alpha \right)}\rangle , \end{array}$$

where 〈〉 denotes average. In our initial trial training runs, we noticed that the loss function always reached a plateau by 800 epochs, so we trained the NNs over 800 epochs for all runs reported in this paper. Because the training is stochastic, the loss function can increase as well as decrease between epochs as seen in Figure 4. At the end of 800 epochs we adopted the network configuration with the lowest loss function regardless of the epoch at which it was achieved.

**Table 2:** Trained models with their corresponding hyperparameters.

**Model**	**Weight (w)**	**p_1_**	**p_2_**	**D_1_*D*_2_*D*_3_*D*_4_**	**Loss**	**〈res〉 (**10^−3^**)**	**99-P res (**10^−2^**)**	**Max res**
								
1	1000	1	1	0.30.30.30.3	MSE	2.77	2.26	0.35
2	1	1	1	0.30.30.30.3	MSE	2.91	2.25	0.37
3	1	1	1	0.40.40.10.1	MSE	3.49	2.03	0.34
4	1	0	1	−−0.30.3	MSE	2.49	1.97	0.38
5	1	0	1	−−0.10.1	MSE	2.04	1.89	0.35
6	1	1	0	0.30.3−−	MSE	75.8	16.5	0.47
7	1	1	0	0.40.4−−	MSE	79.9	21.6	0.65
8	100	1	1	0.30.30.30.3	MAE	2.62	2.59	0.33
9	100	1	1	0.40.40.10.1	MAE	2.08	2.02	0.30
10	1	1	1	0.30.30.30.3	MAE	3.19	3.53	0.40
11	1	1	1	0.40.40.10.1	MAE	2.36	2.66	0.25
12	1	0	1	−−0.10.1	MAE	2.12	2.17	0.34
13	10	0	1	−−0.30.3	MAE	3.15	3.39	0.36
14	10	0	1	−−0.10.1	MAE	2.30	2.46	0.33

*Each model is numbered for reference. The weight w is defined in Equation (1). The D_i_ for i = 1, …, 4 are the dropout values (see Table 1). D_1_ and D_2_ apply to NN 1 whereas D_3_ and D_4_ apply to NN 2. p_1_ and p_2_ are Boolean variables. p_i_ = 0 (p_i_ = 1) implies NN i is turned off (on). If p_1_ = 0 then the values of D_1_ and D_2_ are irrelevant, while p_2_ = 0 makes the values of D_3_ and D_4_ irrelevant. The loss column specifies the loss function, either MSE for mean squared error (α = 2) or mean absolute error MAE (α = 1), respectively (see Equation 1). The mean res, 99-P res and max res columns show the mean, 99-percentile and maximum residual for each model computed over the test set*.

While the trendline (averaged over 5 or 10 epochs) of the loss function value tends to decrease during training, the stochasticity of the training means that the value of the loss function often increases significantly between successive epochs, even by one or two orders of magnitude (see Figure 4). In some cases, the loss function decreases back to its trend after one or two epochs, in other cases (which we call jumps), it stays at the higher value, resetting the trend line to the higher value and only gradually begins to decrease afterwards. In this case all of the epochs after the jump have larger loss functions than the epoch immediately before the jump, as shown for the evolution of the loss function for a typical training run in Figure 4A. This behavior indicates that the stochastic optimization algorithm has pursued an unfavorable branch. To avoid this problem, we added a *roll-back* algorithm to the training, as proposed in Geoffrey (2020). We set a loss threshold value, $L_{thrs}$, such that if the ratio of loss value from epoch *n* to *n*+1 is larger than $L_{thrs}$, then the training algorithm reverts (rolls back) to the NN state corresponding to epoch *n*−*s* and tries again. The stochasticity of training means that roll-back has an effect similar to training an ensemble of models with the same hyperparameters and selecting the model with the lowest loss function value, however, the roll-back optimization takes much less computer time than a large ensemble. We set *s* = 5 and set the threshold value $L_{thrs}$ to

(3) $$\begin{array}{r} L_{thrs}=C\frac{1}{m}\overset{n}{\underset{ep=n-m+1}{\sum }}L^{\left(\alpha \right)}\left(ep\right). \end{array}$$

Here we chose *C* = 5 and *m* = 20 where *ep* stands for epoch, i.e., we set the threshold value to 5 times the average loss function value over the previous *m* = 20 epochs. We chose these values empirically. In Figure 4B, we have plotted a typical example of the evolution of the loss function during training when we train using roll-back. A typical number of roll-backs is 40, i.e., this number is the number of epochs where the jump was higher than the threshold during the training.

## 3. Results

**Figure 5:**
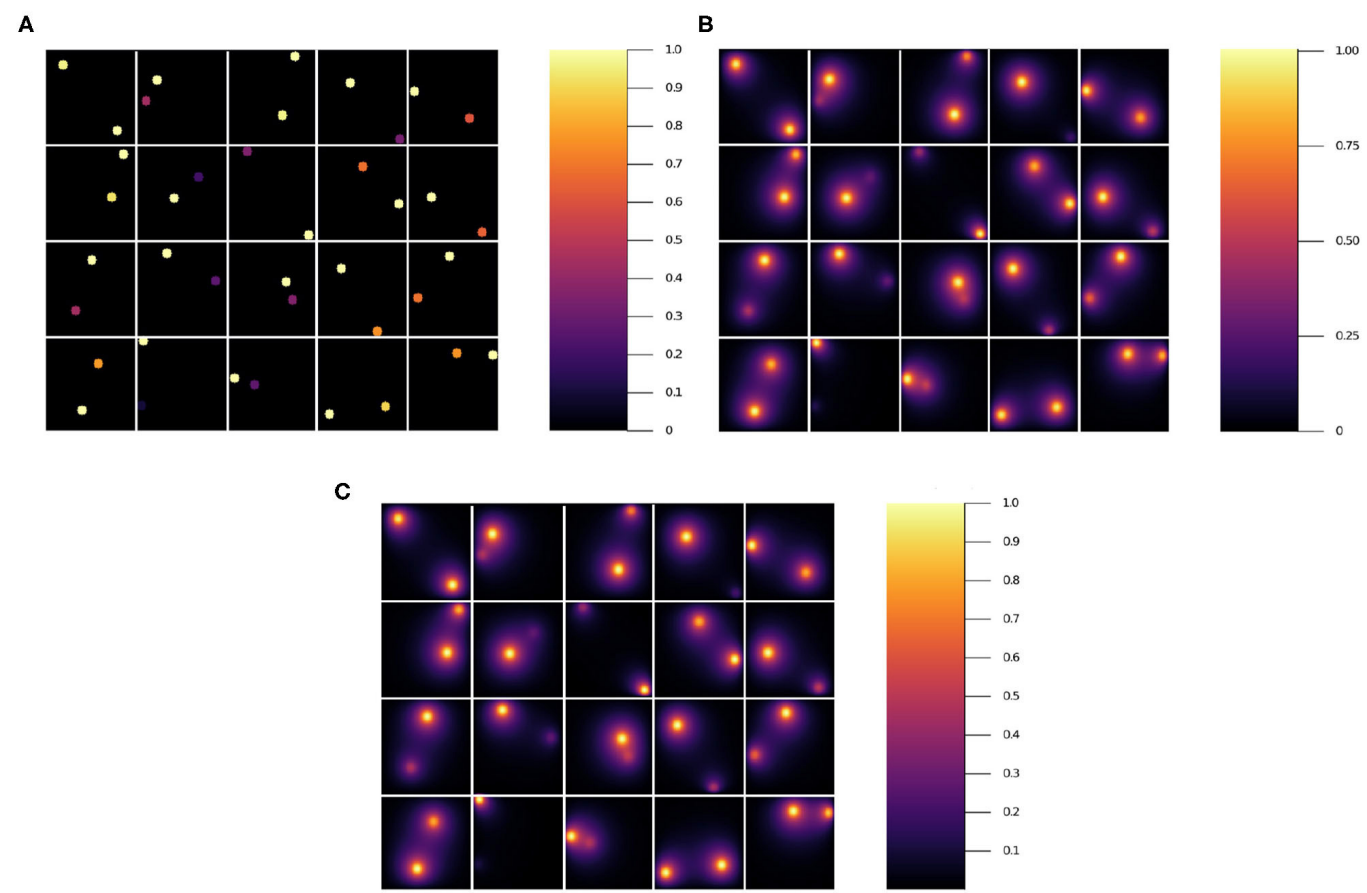
Results for 20 randomly selected test data sets'. **(A)** input, **(B)** ground truth (target output), and **(C)** NN surrogate prediction of steady-state diffusion field output for the input.

**Figure 6:**
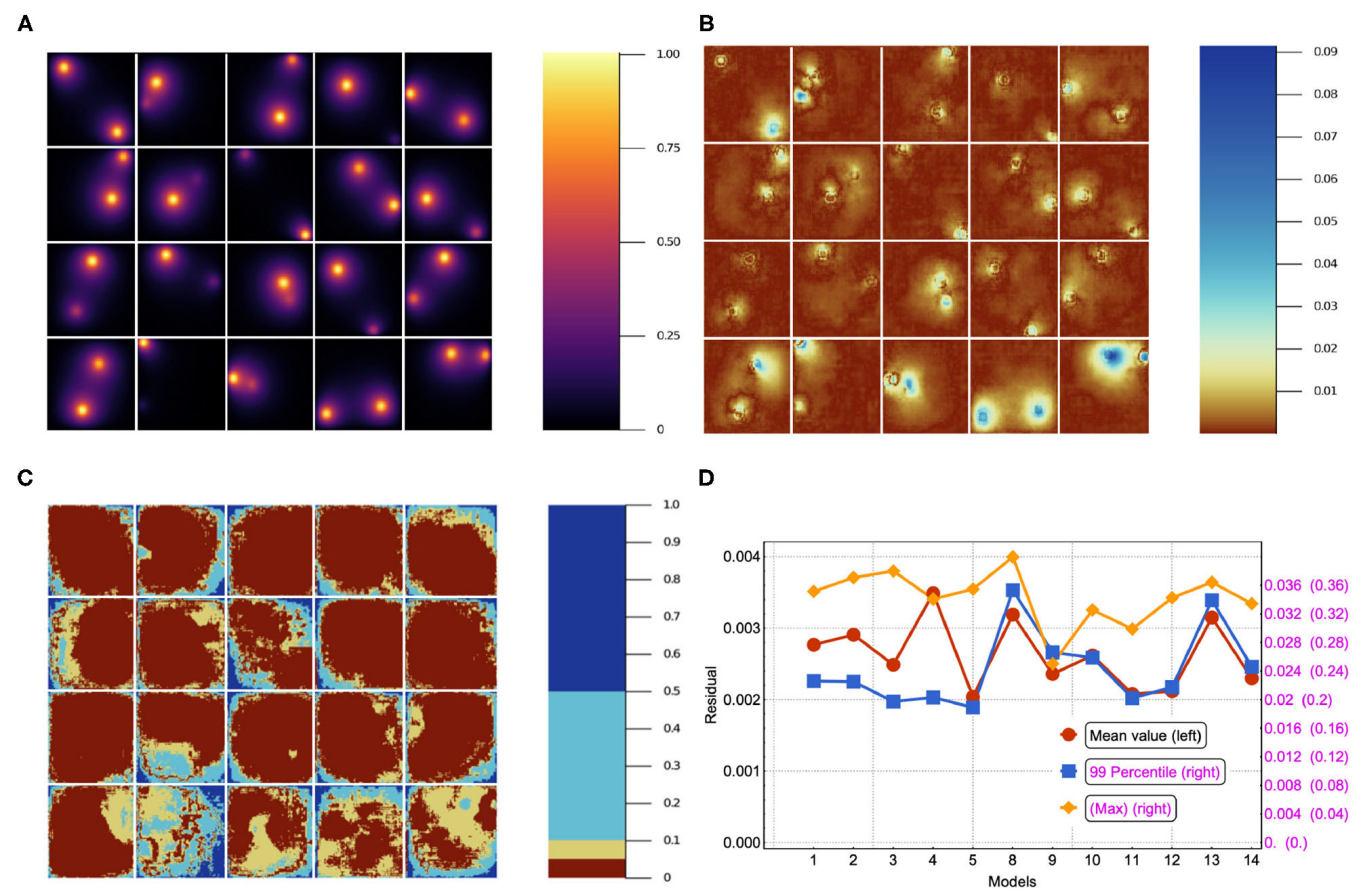
**(A)** The stationary solution for the same batch in the test set. **(B)** Residual (absolute error, i.e., ||*y*_β_〉−|ŷ_β_〉|) for 20 sample source images in the test set trained using model 12 in Table 2. **(C)** Residual/true value (relative error) for the corresponding images. **(D)** Mean, 99-Percentile, and maximum residual for all of the models in Table 2. Left scale for mean value, right scale for 99-Percentile residual value and right scale in parentheses for max residual value.

Quite commonly, the mean residual is the estimator used to judge the goodness of a given model. However, there are cases where the worst predictions are highly informative and can be used to make basic decisions about which features of the NN do not add value. In Figures 5A–C we show 20 different inputs, targets and predictions, respectively. The predictions in Figure 5C were obtained using model 12 (see Table 2) and qualitatively show very good results. For each model we computed the residual, i.e., the absolute value of the difference between the ground truth and the NN prediction pixel-by-pixel, as shown in Figure 6B. We also analyzed the relative residual, *i.e*., the residual divided by the ground truth pixel-by-pixel, as shown in Figure 6C. Models 6 and 7, which only use NN 1 (*p*_1_ = 1 and *p*_2_ = 0), yield mean residuals an order of magnitude larger than models that use both or only NN 2. Therefore, we reject the NN 1-only models and do not analyze them further.

Table 2 summarizes the hyperparameter values for each model we trained. The choice of these parameters was empirically driven. Since we had the field values bounded between 0 and 1 similar to black and white images, we tested different *L*-norms, namely, mean absolute value (MAE), mean squared value (MSE), and mean to the fourth power, often used in neural networks applied to images. In this paper we show the results for MAE and MSE. We also tested different hyperparameters values for the dropout. We found that low dropout values for NN 2 yield the best results.

In Figure 6D, we have plotted the mean residual value, the 99-Percentile residual value and the maximum residual value computed over the test set. Notice that the 99-Percentile residual value is ten times the mean residual value and the maximum residual value is 10 times the 99-Percentile residual value. This suggests that the residual distribution contains outliers, i.e., there is a 1% residual that deviate from mean residual 10 to 100 times. Furthermore, these outliers correspond to regions between the source and the border, near the source, where the source is close to the border as suggested by Figure 6B. While the largest values in absolute residual come from pixels near the source as shown in Figure 6B, the relative error near the source is small whereas the relative error near boundaries is large, as shown in Figure 6C. In Figure 6A we show the stationary solution for the same batch shown in Figures 6B,D. Since we are considering absorbing boundary conditions, the field at the boundary is always equal to zero, thus strictly speaking the relative residual value has a singularity at the boundary. Thus, at the boundaries there is a larger relative error due to the boundary conditions. Since our method has a small absolute error independent of the mean value, the relative error is a poor measure of accuracy for small mean values, since it diverges as the mean approaches zero. Since we have zero-value boundary conditions, at the boundaries there is a larger relative error due to the boundary conditions and therefore the relative error is not a functionally meaningful measure of error unless the system being modeled is highly sensitive to small values of the field. Oxygen levels in normal tissues fluctuate significantly in space and time. For instance, in the retina, oxygen concentration fluctuates dramatically in space, time and depending on illumination. Short-term temporal fluctuations range from 5 to 50% depending on depth in cat retina (Linsenmeier and Zhang, 2017). This intrinsic oxygenation fluctuation in tissues suggests that biologically, 5% relative error at low concentrations is an acceptable accuracy for oxygen concentration estimation.

Models 5, 11, and 12 have low mean residuals with model 5 being the smallest. Focusing instead on the mean residual and the 99-Percentile, we notice that models 3, 4, 5, 11, and 12 yield the best results. Finally, considering the maximum residual together with the previous estimators, we notice that model 9 has low mean residual, low 99-percentile residual and the lowest max residual. Depending on the user's needs, one estimator will be more relevant than others. In this sense, defining a *best* model is relative. Nevertheless, having more metrics (e.g., relative error for large values and absolute error for small values) helps to characterize each model's performance. In future work we'll consider more adaptable metrics, as well as mixed error functions that incorporate multiple estimators.

Figure 8 plots the prediction vs. the target for each pixel in each image in the training and test sets for models 9 and 11. Notice that for the test sets the results are qualitatively similar between models, for the training set the dispersion is larger in model 11 than in model 9. This suggests model 11 is overfitting the training data. Models 9 and 11 have the same hyperparameters except for the weight *w*. In the former *w* = 100 while in the latter *w* = 1. This suggests that the exponential weight helps reduce overfitting.

**Figure 7:**
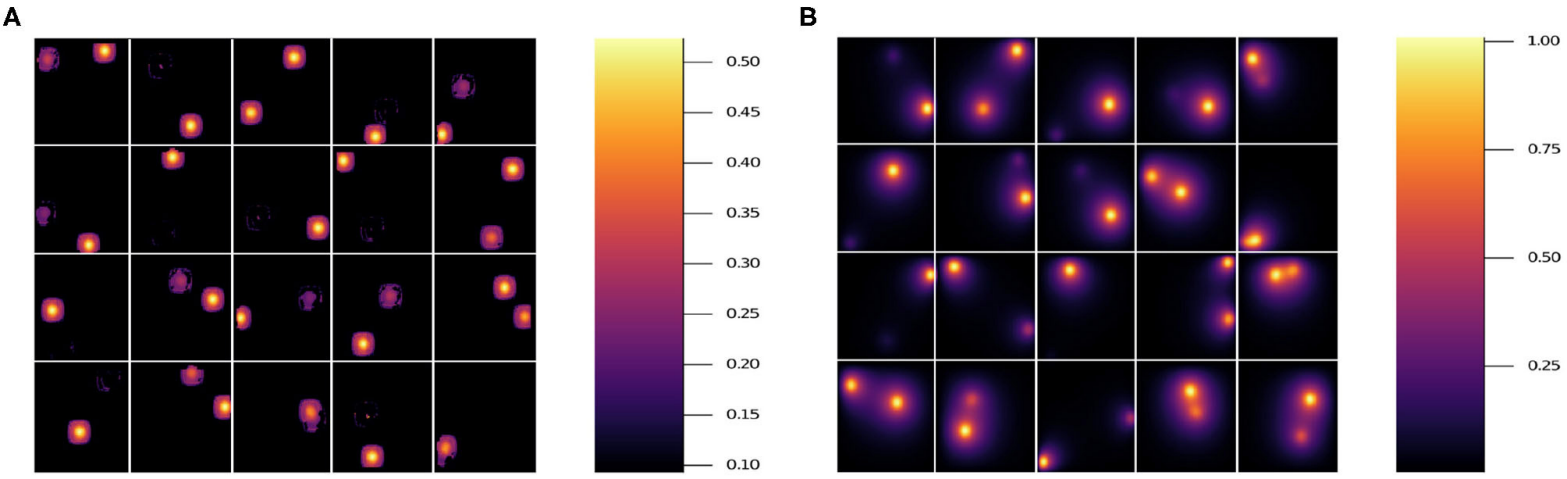
Results for 20 randomly selected test data sets'. **(A)** Prediction using model 7, which only uses NN 1. **(B)** Prediction using model 5, which only uses NN 2 (see Table 2). Note the different scale on the color bars.

**Figure 8:**
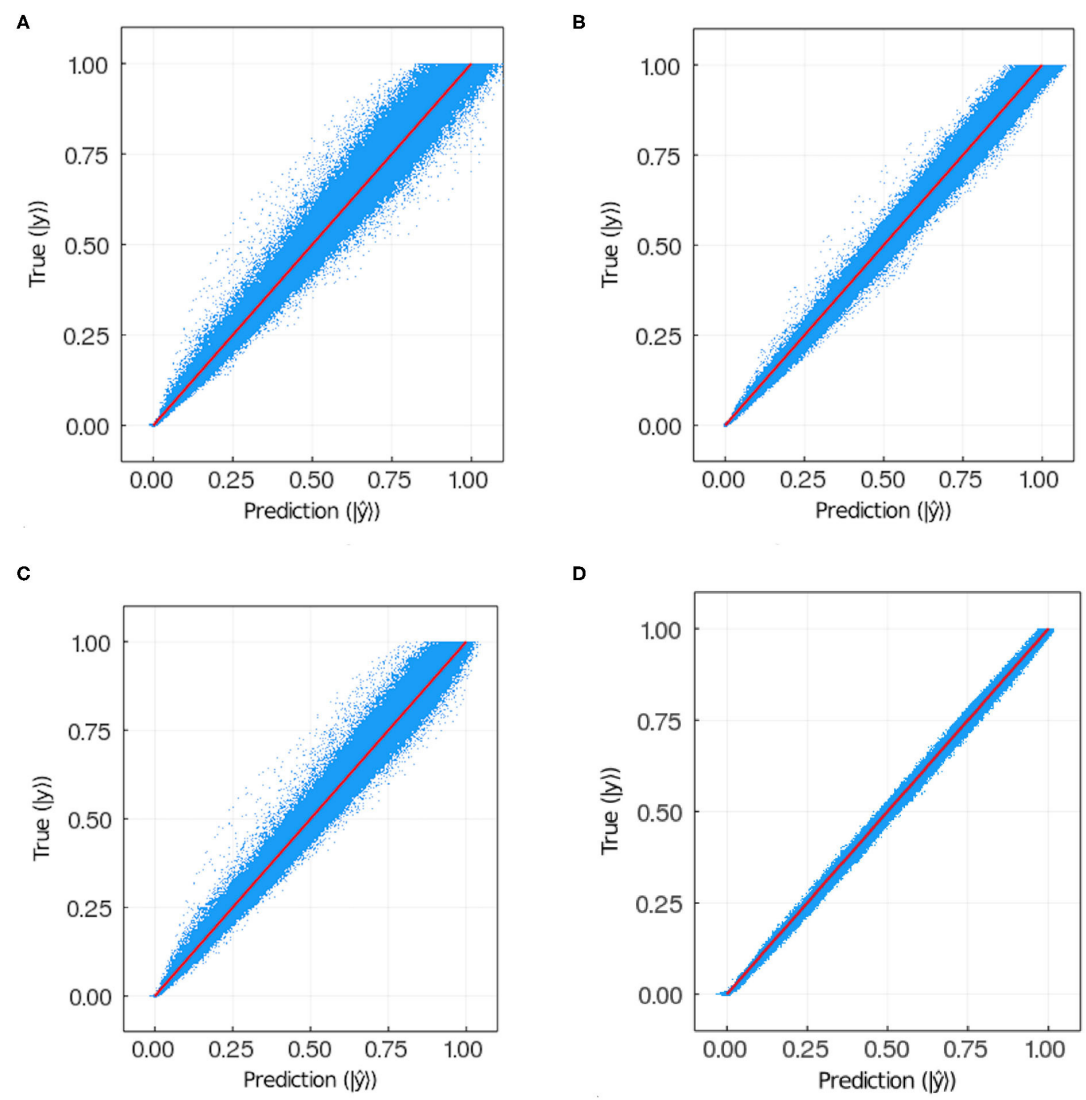
Ground truth vs. prediction for **(A)** test set and **(B)** training set in the case of model 9; **(C)** test set and **(D)** training set in the case of model 11 (see Table 2). The number of points plotted in each panel is 3.75·10^7^.

**Figure 9:**
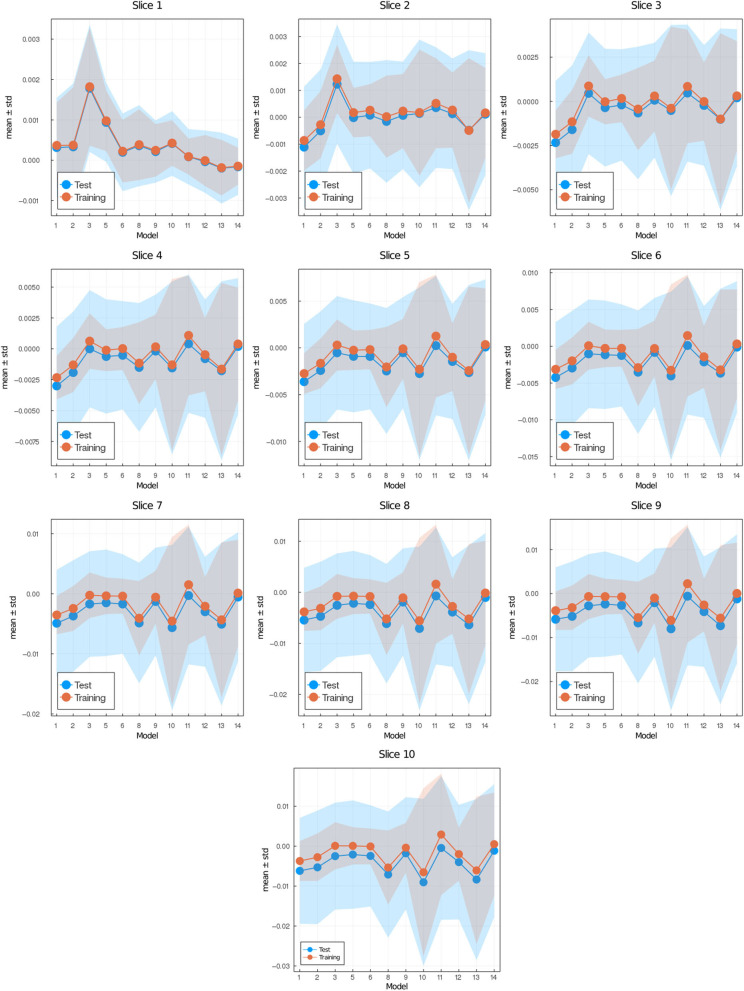
Mean (data points) ± standard deviation (envelop) per slice vs. models (see Table 1) for test set (blue) and training set (red). Slice *i* corresponds to field values in the interval [0.1·(*i*−1), 0.1·*i*] where *i* = 1, …, 10.

In Figure 7, we show the prediction from NN 1 (Figure 7A) and NN 2 (Figure 7B). Notice that NN 1 is able to detect the sources whereas NN 2 is able to predict the field. Using both neural networks improves the results as can be seen in Figure 6D. As previously mentioned, pixels with low (near 0) field values are much more common than pixels with high (near 1) field values. While the exponential factor in the loss function compensates for this bias, the residual in Figure 6D does not. To address this issue we compute the mean residual over small field intervals. This will tell us how well the model predicts for each range of absolute values. Furthermore, this method can be used to emphasize accuracy or relative accuracy in different value ranges. The way we do this is as follows. In Figure 8, we take 10 slices of size 0.1 in the direction *y* = *x*. We then compute the mean residual and standard deviation per slice. In Supplementary Material (section 1), we have plotted the PDF (probability density function) per slice (blue bins) and a Gaussian distribution (red curve) with mean and standard deviation set to the mean residual and standard deviation per slice, respectively. We did this for all models in Table 2. In Figure 9, we plotted the mean residual vs. for each model for each slice for the test and training sets. The error envelop shows the residual standard deviation per slice. Notice that models trained with MSE have a smaller residual standard deviation than models trained with MAE in the case of the training set, which suggest that MSE contributes to overfitting more that MAE. Recall that the difference between the MSE gradient and the MAE gradient is that the former is linear with the residual value whereas the latter is a constant. Therefore, training with MAE generalizes better than MSE. Additionally, notice the dispersion increases with the slice number.

**Figure 10:**
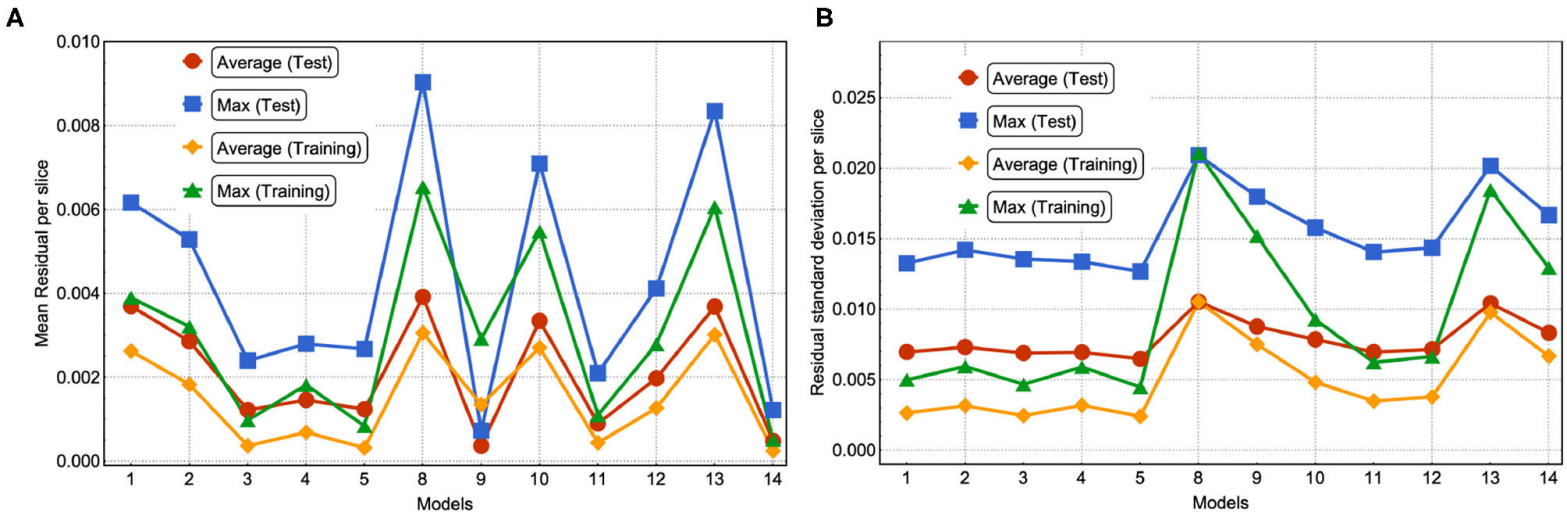
**(A)** For each model, we show the average and maximum over the residual mean value per slice. **(B)** For each model, we show the average and maximum over the residual standard deviation per slice (see Figure 9). This was done for the test and training set.

**Figure 11:**
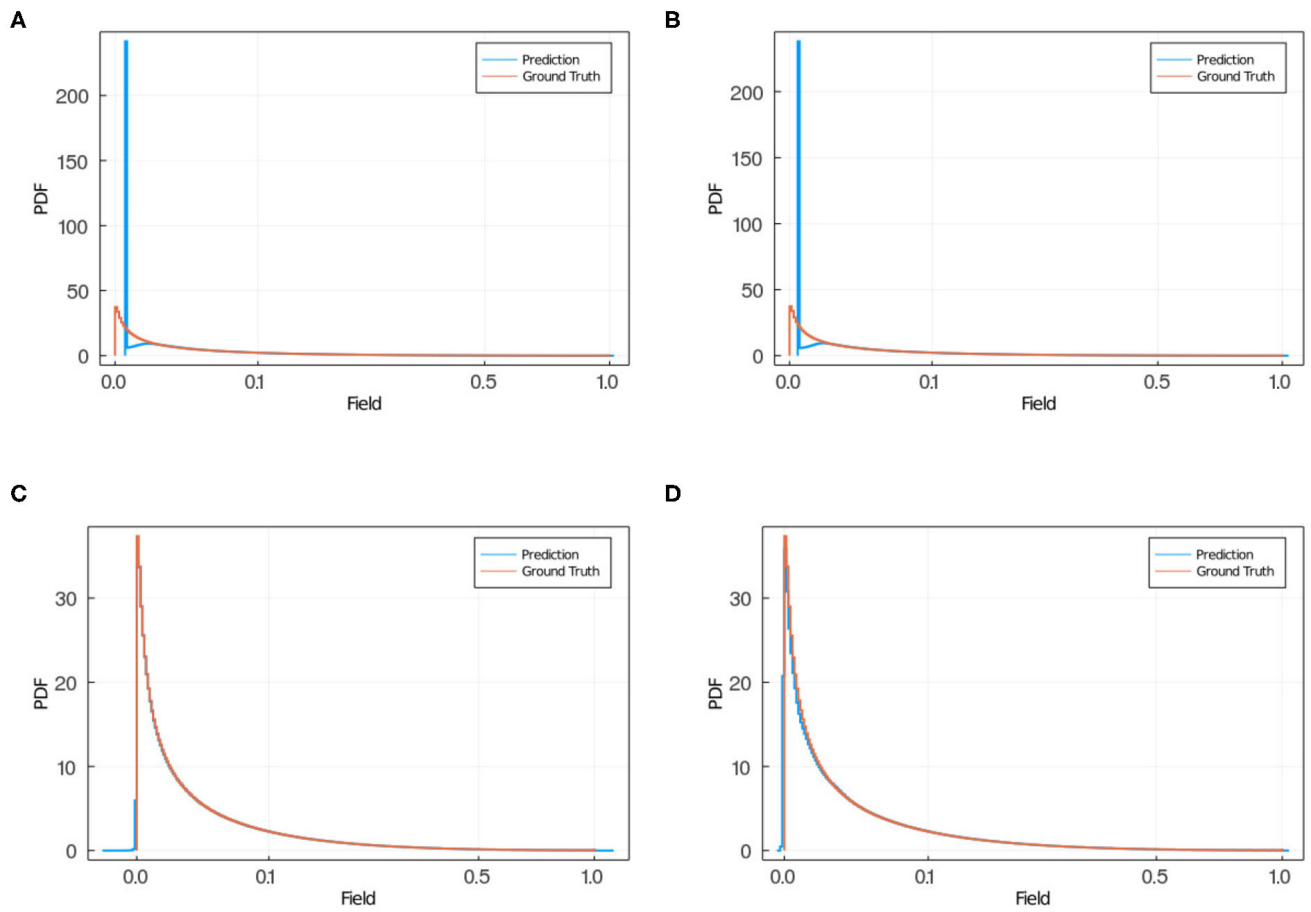
PDF of field obtained via NN (blue) and ground truth (red) in the case of training using MSE, for **(A)** model 2 and **(B)** model 3 and for training using MAE, for **(C)** model 11 and **(D)** model 12. When using MSE **(A,B)** the NN predicts zero field values instead of low non-zero field values as the predicted PDF has a larger peak in zero than the ground truth PDF, and a smaller PDF for small non-zero field values compared with the ground truth PDF. When training using MAE **(C,D)** the prediction and ground truth PDFs overlap completely.

In Figure 10, we plotted the average and maximum over the residual mean value per slice (see Figure 10A) and the residual standard deviation per slice (see Figure 10B) for each model's test and training sets. Notice that in this approach, by slicing the residual values and computing the average residual over the set of slices, we are giving equal weight to each mean residual per slice and, therefore, compensating for the imbalance in frequency of low and high value pixels. An interesting feature from using MSE or MAE comes from the PDF of the field values. Training using MAE makes the PDF prediction quite accurate as the prediction completely overlaps with the ground truth (see Figure 11). In comparison, when training with MSE, the PDF is not as good and the overlap between ground truth and prediction is not complete. There is a mismatch for low field values in the sense that the NN does not predict low non-zero field values correctly. Thus, we recommend using MAE to avoid this issue.

## 4. Discussion

In large-scale mechanistic simulations of biological tissues, calculations of the diffusion of molecular species can be a significant fraction of the total computational cost. Because biological responses to concentrations often have a stochastic overlay, high precision may not be essential in these calculations Because NN surrogate estimates are significantly faster than the explicit calculation of the steady-state diffusion field for a given configuration of sources and sinks, an effective NN surrogate could greatly increase the practical size of simulated tissues, e.g., in cardiac simulations (Kerckhoffs et al., 2007; Sundnes et al., 2014), cancer simulations (Bruno et al., 2020), and orthopedic simulations (Erdemir et al., 2007). In our case, using a NVIDIA Quadro RTX 6000, each diffusion solution is about 1,000 times faster using the trained NN solver compared to the Julia code.

In order to decide if this acceleration is useful, we have to consider how long it takes to run the direct simulation, how long the NN takes to train and how long it takes to execute the NN once it has been trained (Fox and Jha, 2019). If each diffusion calculation takes δ seconds to run, conducting *N* calculations directly takes *t*_*direct*_ = *Nδ*. If each neural network surrogate takes ϵ seconds to run, and the number of replicas in the training set is *M* and the training time is *E*, the total time for the neural network simulation is the time to generate the training set, the training time plus the simulation time, *t*_*neuro*_ = *Mδ*+*E*+*Nϵ*. To estimate these times, we ran 20, 000 explicit simulations in Julia, which took ~ 6 h and 30 min, yielding roughly 1.16s each. The NN training time was 12 h on average. While the speedup for an individual simulation is δ/ϵ≈1, 000, the ratio τ_*neuro*_/τ_*direct*_ must be smaller than 1 in order to have a useful acceleration. Equating this ratio to 1 and solving for *N* yields

(4) $$\begin{array}{r} N_{min}=\frac{M+E/\delta }{1-\epsilon /\delta }\approx M+\frac{E}{\delta }. \end{array}$$

*N*_*min*_ gives the number of replicas necessary for the total time using the NN to be the same as the direct calculation. Of course, the exact times will depend on the specific hardware used for the direct and NN calculations. In our case, from Equation (4) we obtain that *N*_*min*_≈57, 300, we would need to use the neural network more than 57, 300 times for the total time using the NN to be faster than the direct calculation. Thus the NN acceleration is primarily useful in simulations that will be run many, many times for the specific situation for which the NN is appropriate. Consider for example if one wishes to include a variable number of sources, different lattice sizes, different dimensionalities (e.g., 3D) and boundary conditions. The more general the NN the more training data it will require, the longer training will take, and the slower the individual NN calculations will be. Currently virtual-tissue simulation studies often run thousands to tens of thousands of replicas and each replica often takes tens of minutes to tens of hours to run. This computational cost makes detailed parameter identification and uncertainty quantification impractical, since simulations often have dozens of parameters to explore. If using a NN-based diffusion solver accelerated these simulations by 100 × it would permit practical studies with hundreds of thousands to millions of replicas, greatly expanding the feasible exploration of parameter space for parameter identification and uncertainty quantification. It is worthwhile mentioning that there are other numerical methods for diffusion in 2D and 3D models that can also exploit the GPU parallelization such as the one in Secomb (2016) based on a discretization of Green's function. Our focus is on the ability of neural-network surrogates to solve the time-independent diffusion equation, however it would be interesting to extensively optimize the mechanistic methods we used to generate our training data sets. Generating training data is so time consuming, applications of deep neural networks will benefit greatly from using faster mechanistic methods to generate training data.

While there isn't a protocol for setting up a diffusion-solver surrogate, there are several things that must be considered. First one needs to frame the problem similar to how one would do when performing mechanistic modeling. One needs to settle on the dimensionality, e.g., 1D, 2D, 3D,… or *n*-dimensions; the system size which in our case we settled on 100 × 100; the type of sources to consider, e.g., sinks, sources, or both; the boundary conditions e.g., absorbing, reflective, periodic, or mixed; the distribution of sources in space; and it is also important to think about the accuracy required from the neural network. There isn't a rigorous way to determine the size of the required training dataset, although the size will depend on the problem one is addressing and the decisions made in the previous step. We recommend to start with a training dataset size of the order ~ 10^4^. Then one needs to decide on the network architecture. For the network architecture the number of options is large. For instance, the depth of the neural network, e.g., deep or shallow; the type of layers, e.g., convolutional layers, fully-connected layers, recurrent layers, or mixed layers; the activation functions, e.g., ReLU, sigmoid, tanh, etc. Evidence suggests that using deep neural networks as opposed to shallow neural networks will increase the non-linearity of the neural network, which ultimately broadens the learning capabilities of the neural network (Bianchini and Scarselli, 2014). But as depth increases the gradient of the loss function can grow or diminish significantly leading to instabilities or a regime of *zero learning* where the gradient becomes zero but the loss function value is large. Very deep neural networks can also lead to an *overflow* or *underflow* situation. Therefore, the neural network depth is a feature that should be set in way that meets a middle ground. The right choice of activation functions, regularizer layers (i.e., DropOut, BatchNorm, etc.) and weight initializers can hinder the unwanted features of instabilities or *zero learning*. Choosing the right optimizer for training is also something to consider. However, unless there are some very specific needs, the standard rule is to use the ADAM optimizer (Kingma and Ba, 2014). Choosing the loss function is crucial as this is the metric the neural network will use to measure how *good* the outcome is. Typically for these type of problems MSE, MAE and other similar norms are used. Additionally, there are a number of (hyper)parameters to be chosen. For instance, some activation functions, regularizer layer and optimizers have hyperparameters. Also the number of epochs and size of minibatch are hyperparameters. To set the hyperparameters' values, one can start by using the values reported in the literature but the scope should be to explore the space of hyperparameters by training an ensemble of neural networks with different hyperparameters and then choosing the model that performed best on the validation set.

In a real tissue, the oxygen tension on the surface of the blood vessel and in the tissue as a whole involves complex feedback among many factors, including spatial and temporal variation in the supply and consumption of oxygen; supply at a given location could depend on the degree of local blood-vessel dilation, the rate of blood flow, and levels of oxygen in the blood to name a few examples. A realistic model of oxygenation in tissue would need to include spatial and temporal models of all of these processes individually and of their coupling. Clearly such a model is much more complex than our simple example of calculating the steady-state oxygen field given a fixed set of circular sources with fixed oxygen tensions and a fixed uniform consumption rate in the tissue implemented as linear decay.

While developing NN surrogates to solve the entire complex problem of oxygenation would be worthwhile, we believe that deep neural network surrogates will (at least initially) not replace the entire simulation, but to replace the most computationally costly components of the simulation. In this case, looking for surrogates for specific commonly-used calculations, which can be used in many different applications and which can provide a substantial speed-up is appropriate. Many biophysical and engineering problems require solving the diffusion equation for fixed sources. Despite the improvements to direct solution mentioned in Secomb (2016), solving the diffusion equation still often contributes much of the computational cost of the full problem solution. In these cases, the faster the “diffusion step” is computed, the faster the solution of the multiscale model as a whole. To train an optimal diffusion surrogate for a particular problem one has to choose a set of appropriate loss functions and combine them to minimize the errors of the metrics one defines as most relevant to the specific problem being addressed. How to choose loss functions and their weighting to achieve macroscopic desired outcomes is not well understood as a general problem. Even in our very simple example, we had to explore a wide variety of loss functions to achieve reasonable convergence of our NN during training and reasonable final absolute and relative accuracy of our surrogate.

## 5. Conclusions

Neural networks provide many possible approaches to generating surrogate diffusion solvers. Given the type of problem setting, we were interested in a neural network that could predict the stationary field. We considered a deep convolutional neural network, an autoencoder and their combination. We considered two loss functions, *viz*. mean squared error and mean absolute error. We considered different hyperparameters for dropout and an exponential weight to compensate the under-sampling of high field values. The exponential weight also helped reduce overfitting as shown in Figure 8.

The range of scientific and engineering applications for diffusion solvers is very broad. Depending on the specific application, the predictions by the neural network will have to meet a specific set of criteria quantified in the form of statistical estimators (e.g., mean error, max error, percentiles, mean relative error, *etc*.). In this paper we studied several reasonable error metrics, namely, mean residual, maximum residual, 99-Percentile residual, mean relative residual, mean weighted residual and the weighted standard deviation residual. The last two metrics compensate for the low frequency of high field values, ones that usually occur in small regions around sources. The autoencoders are commonly used in generative models which is applicable, as we have shown here, to the case of a diffusion surrogate. The field predictions are accurate on all the metrics we considered. This is appears to be due to collapsing the input into a one-dimensional vector and then decoding back to the initial size, which forces the network to learn the relevant features (Kingma and Welling, 2019). While some models had high errors across all metrics, no single model had the smallest error for all error metrics. Different networks and hyperparameters were optimal for different metrics, e.g., model 5 had the lowest mean residual, whereas model 9 yielded relatively good results on all metrics. Model 9 uses both neural networks with the dropout values for the deep convolutional network were set to *D*_1,2_ = 0.4, and for the autoencoder to *D*_3,4_ = 0.1. The weight hyperparameter was set to 100. Recall that large weight hyperparameter values make the loss function weight high field values over low field values. This is important since the largest absolute error happens close to sources and close to boundaries because of the under-representation of these kinds of configurations. We also noticed that this choice reduced the overfitting as was shown in Figure 8.

Additionally, we tested several loss function. Here we reported the results using mean squared error and mean absolute error. We noticed two key differences. With MSE the weighted standard deviation (see Figure 9) is smaller than for MAE for the training set. However, for the test set, the results for both loss functions are comparable. This difference between training and test sets suggests that MSE is more prone to overfitting the data than MAE. The other key difference is that for the MAE, the predicted field probability function consistently overlapped the ground truth completely, whereas for MSE there is a mismatch in that the NN does not predict low non-zero field values correctly (see Figure 11). Therefore, we recommend using MAE as the loss function for surrogate calculations where the field values are well bounded, as we have shown it produces better predictions than MSE. The autoencoder (NN 2) is capable of approximating the diffusion field on its own, the convolutional network (NN 1) is not. However, if we use the two networks together we find that the prediction is more accurate than NN 2 alone.

These encouraging results suggest that we should pursue NN surrogates for acceleration of simulations in which the solution to the diffusion equation contributes a considerable fraction of the total computational cost. An effective NN diffusion solver surrogate would need to be able to solve diffusion fields for arbitrary sources and sinks in two or three dimensions with variable diffusivity, a much higher dimensional set of conditions than the two circular sources in a uniform two-dimensional square domain that we investigated in this paper. A key question will be the degree to which NNs are able to generalize, e.g., from *n* sources to *n*+1 sources or from circular sources to more complex shapes. In addition, here we only considered absorbing boundary conditions, ultimately mixed boundary conditions are desirable. It is unclear if the best approach would be a single NN capable of doing multiple boundary conditions, or better to develop unique NNs for each boundary condition scenario. While in this paper we have only considered zero-field boundary conditions mainly due to feasibility purposes for the neural network, we will consider different boundary conditions in future work.

Increasing the number and size of vessels is a combinatorial problem in the dimensionality of the training set, but it ultimately doesn't change the nature of the diffusion equation. Thus, we expect that a straightforward approach consisting using a bigger training set including a greater variety of source and sink sizes, shapes, and number, should still work, though it will take more computing time to generate the training data and train the network. The ability of greens-function methods to solve the diffusion equation for arbitrary numbers of sources and sinks suggests (though it does not prove) that such generalization should work also for neural network solvers.

To solve 3D diffusion problems, the most straightforward extension of our method would be to use 3D convolutional neural networks. However, there may be some difficulties with a naive extension of our convolutional methods to 3D. If we have a linear dimension of *L* then the output layer of the NN has *L*^2^ elements in 2D and *L*^3^ in 3D. Thus, for a given value of *L*, the network size is much larger in 3D. Besides the size of the network, the training set will also be larger. For *N* sources, the number of possible configurations grows roughly as *L*^2*N*^ in 2D, while the number of configurations in 3D is *L*^3*N*^. In addition, if we wish to represent realistic sources in 3D, like blood vessels, we need to sample over appropriately spatially-correlated patterns of sources rather than the randomly located spherical sources we used in our 2D example. Naively these very high dimensions of possible source configurations suggest that the 3D problem would require impossibly large training datasets. However, one of the outstanding features of deep neural networks is their capacity to extrapolate from apparently severely undersampled training sets, so increasing the number of possible configurations exponentially does not necessarily imply the need to increase the training set exponentially. Another approach to develop diffusion solver surrogates in 3D is to build physically informed neural networks (PINNs) (Raissi et al., 2019) where the ODE describing the process, the initial conditions and the boundary conditions are embedded in the loss function. Other efforts attempt to tackle the *curse of dimensionality* by physical intuition embedded in the neural network architecture (Roberts, 2021). We will explore these issues in future work.

## Data Availability Statement

The datasets presented in this study can be found in online repositories. The names of the repository/repositories and accession number(s) can be found at: https://github.com/jquetzalcoatl/DiffusionSurrogateDeepLearning.

## Author Contributions

JG and JS proposed the project. JT-M and GF built the models. JT-M trained the models. All authors analyzed the results and wrote the manuscript.

## Conflict of Interest

The authors declare that the research was conducted in the absence of any commercial or financial relationships that could be construed as a potential conflict of interest.

## Footnotes

^1^We use the terms *deep learning* and *machine learning* interchangeably. We also use *neural network* and *deep neural network* interchangeably.

## References

[B1] Baur C. Denner S. Wiestler B. Navab N. Albarqouni S. (2020). Autoencoders for unsupervised anomaly segmentation in brain MR images: a comparative study. Med. Image Anal. 2020:101952. 10.1016/j.media.2020.101952 33454602

[B2] Bianchini M. Scarselli F. (2014). On the complexity of neural network classifiers: a comparison between shallow and deep architectures. IEEE Trans. Neural Netw. Learn. Syst. 25, 1553–1565. 10.1109/TNNLS.2013.2293637 25050951

[B3] Bruno R. Bottino D. de Alwis D. P. Fojo A. T. Guedj J. Liu C.. (2020). Progress and opportunities to advance clinical cancer therapeutics using tumor dynamic models. Clin. Cancer Res. 26, 1787–1795. 10.1158/1078-0432.CCR-19-0287 31871299 PMC8415106

[B4] Cai S. Wang Z. Wang S. Perdikaris P. Karniadakis G. (2021). Physics-informed neural networks (PINNS) for heat transfer problems. J. Heat Transf. 143:060801. 10.1115/1.4050542

[B5] Champion K. Lusch B. Kutz J. N. Brunton S. L. (2019). Data-driven discovery of coordinates and governing equations. Proc. Natl. Acad. Sci. U.S.A. 116, 22445–22451. 10.1073/pnas.1906995116 31636218 PMC6842598

[B6] Chen M. Shi X. Zhang Y. Wu D. Guizani M. (2017). Deep features learning for medical image analysis with convolutional autoencoder neural network. IEEE Trans. Big Data. 1-1. 10.1109/TBDATA.2017.2717439

[B7] Chen R. T. Q. Rubanova Y. Bettencourt J. Duvenaud D. K. (2018). “Advances in neural information processing systems,” in Neural Ordinary Differential Equations, Vol. 31, eds S. Bengio, H. Wallach, H. Larochelle, K. Grauman, N. Cesa-Bianchi, R. Garnett (Curran Associates, Inc.)

[B8] Chen W. Ferguson A. L. (2018). Molecular enhanced sampling with autoencoders: on-the-fly collective variable discovery and accelerated free energy landscape exploration. J. Comput. Chem. 39, 2079–2102. 10.1002/jcc.25520 30368832

[B9] Dubois P. Gomez T. Planckaert L. Perret L. (2020). Data-driven predictions of the lorenz system. Phys. D 2020:132495. 10.1016/j.physd.2020.132495

[B10] Edalatifar M. Tavakoli M. B. Ghalambaz M. Setoudeh F. (2020). Using deep learning to learn physics of conduction heat transfer. J. Therm. Anal. Calorim. 1–18. 10.1007/s10973-020-09875-6

[B11] Erdemir A. McLean S. Herzog W. van den Bogert A. J. (2007). Model-based estimation of muscle forces exerted during movements. Clin. Biomech. 22, 131–154. 10.1016/j.clinbiomech.2006.09.005 17070969

[B12] Farimani A. B. Gomes J. Pande V. S. (2017). Deep learning the physics of transport phenomena. arXiv preprint. arXiv:1709.02432.

[B13] Fox G. Jha S. (2019). “Learning everywhere: a taxonomy for the integration of machine learning and simulations,” in 2019 15th International Conference on eScience (eScience), (San Diego, CA), 439–448. 10.1109/eScience.2019.00057

[B14] Geoffrey F. (2020). Draft Deep Learning for Spatial Time Series. Technical Report. Available online at: https://www.dsc.soic.indiana.edu

[B15] Gkeka P. Stoltz G. Farimani A. B. Belkacemi Z. Ceriotti M. Chodera J.. (2020). Machine learning force fields and coarse-grained variables in molecular dynamics: application to materials and biological systems. arXiv preprint arXiv:2004.06950. 10.1021/acs.jctc.0c00355 PMC8312194 32559068

[B16] He H. Pathak J. (2020). An unsupervised learning approach to solving heat equations on chip based on auto encoder and image gradient. arXiv preprint arXiv:2007.09684.

[B17] Innes M. (2018). Flux: elegant machine learning with Julia. J. Open Source Softw. 3:602. 10.21105/joss.00602

[B18] Innes M. Saba E. Fischer K. Gandhi D. Rudilosso M. C. Joy N. M.. (2018). Fashionable modelling with flux. CoRR, abs/1811.01457.

[B19] Kasim M. Watson-Parris D. Deaconu L. Oliver S. Hatfield P. Froula D. H.. (2020). Up to two billion times acceleration of scientific simulations with deep neural architecture search. arXiv preprint arXiv:2001.08055.

[B20] Kerckhoffs R. C. Neal M. L. Gu Q. Bassingthwaighte J. B. Omens J. H. McCulloch A. D. (2007). Coupling of a 3d finite element model of cardiac ventricular mechanics to lumped systems models of the systemic and pulmonic circulation. Ann. Biomed. Eng. 35, 1–18. 10.1007/s10439-006-9212-7 17111210 PMC2872168

[B21] Kingma D. P. Ba J. (2014). Adam: Amethod for stochastic optimization. arXiv preprint arXiv:1412.6980.

[B22] Kingma D. P. Welling M. (2019). An introduction to variational autoencoders. arXiv preprint arXiv:1906.02691. 10.1561/9781680836233

[B23] Lee Y. Veerubhotla K. Jeong M. H. Lee C. H. (2020). Deep learning in personalization of cardiovascular stents. J. Cardiovasc. Pharmacol. Therap. 25, 110–120. 10.1177/1074248419878405 31554426

[B24] Li A. Chen R. Farimani A. B. Zhang Y. J. (2020). Reaction diffusion system prediction based on convolutional neural network. Sci. Rep. 10, 1–9. 10.1038/s41598-020-60853-2 32127569 PMC7054402

[B25] Li Z. Kovachki N. Azizzadenesheli K. Liu B. Bhattacharya K. Stuart A.. (2020). Fourier neural operator for parametric partial differential equations. arXiv preprint arXiv:2010.08895.

[B26] Linsenmeier R. A. Zhang H. F. (2017). Retinal oxygen: from animals to humans. Prog. Retinal Eye Res. 58, 115–151. 10.1016/j.preteyeres.2017.01.003 PMC5441959 28109737

[B27] Noé F. Olsson S. Köhler J. Wu H. (2019). Boltzmann generators: sampling equilibrium states of many-body systems with deep learning. Science 365:eaaw1147. 10.1126/science.aaw1147 31488660

[B28] Noé F. Tkatchenko A. Müller K.-R. Clementi C. (2020). Machine learning for molecular simulation. Annu. Rev. Phys. chem. 71, 361–390. 10.1146/annurev-physchem-042018-052331 32092281

[B29] Phillips R. (2018). “Membranes by the numbers,” in Physics of Biological Membranes, eds P. Bassereau and S. Pierre (Springer), 73–105. 10.1007/978-3-030-00630-3_3

[B30] Rackauckas C. Innes M. Ma Y. Bettencourt J. White L. Dixit V. (2019). Diffeqflux.jl - A julia library for neural differential equations. CoRR, abs/1902.02376.

[B31] Rackauckas C. Nie Q. (2017). Differentialequations.jl-a performant and feature-rich ecosystem for solving differential equations in Julia. J. Open Res. Softw. 5:15. 10.5334/jors.151

[B32] Raissi M. Perdikaris P. Karniadakis G. E. (2019). Physics-informed neural networks: a deep learning framework for solving forward and inverse problems involving nonlinear partial differential equations. J. Comput. Phys. 378, 686–707. 10.1016/j.jcp.2018.10.045

[B33] Roberts D. A. (2021). Why is AI Hard and Physics Simple? Technical report. arxiv preprint. arXiv:2104.00008

[B34] Schiesser W. E. (2012). The Numerical Method of Lines: Integration of Partial Differential Equations. San Diego, CA: Elsevier.

[B35] Secomb T. W. (2016). A green's function method for simulation of time-dependent solute transport and reaction in realistic microvascular geometries. Math. Med. Biol. 33, 475–494. 10.1093/imammb/dqv031 26443811 PMC5155623

[B36] Sharma R. Farimani A. B. Gomes J. Eastman P. Pande V. (2018). Weakly-supervised deep learning of heat transport via physics informed loss. arXiv preprint arXiv:1807.11374.

[B37] Sundnes J. Wall S. Osnes H. Thorvaldsen T. McCulloch A. D. (2014). Improved discretisation and linearisation of active tension in strongly coupled cardiac electro-mechanics simulations. Comput. Methods Biomech. Biomed. Eng. 17, 604–615. 10.1080/10255842.2012.704368 22800534 PMC4230300

[B38] Tikhonov A. N. Samarskii A. A. (2013). Equations of Mathematical Physics. New York, NY: Courier Corporation. Available online at: https://books.google.ca/books?hl=en&lr=&id=PTmoAAAAQBAJ&oi=fnd&pg=PP1&dq=Tikhonov+and+Samarskii,+2013&ots=kYk8H8xNep&sig=pNN3S9TRC0RhHhlZYdobzBWT66Y&redir_esc=y#v=onepage&q=Tikhonov%20and%20Samarskii%2C%202013&f=false

[B39] Toledo-Marin J. Q. (2020). Stationary Diffusion State Ml Surrogate Using Flux and Cuarrays. Available online at: https://github.com/jquetzalcoatl/DiffusionSurrogate (accessed June 09, 2021).

[B40] Xie D. Li Y. Yang H. Song D. Shang Y. Ge Q.. (2019). “Bold fMRI-based brain perfusion prediction using deep dilated wide activation networks,” in International Workshop on Machine Learning in Medical Imaging (Shenzhen: Springer), 373–381. 10.1007/978-3-030-32692-0_43

[B41] Zhang H. Hippalgaonkar K. Buonassisi T. Løvvik O. M. Sagvolden E. Ding D. (2019). Machine learning for novel thermal-materials discovery: early successes, opportunities, and challenges. arXiv preprint arXiv:1901.05801. 10.30919/esee8c209

[B42] Zhang J.-S. Xiao X.-C. (2000). Predicting chaotic time series using recurrent neural network. Chinese Phys. Lett. 17:88. 10.1088/0256-307X/17/2/004

