# Supplementary material for: Deep Learning Approaches to Surrogates for Solving the Diffusion Equation for Mechanistic Real-World Simulations
Source: Front Physiol. 2021 Jun 24;12:667828. doi: 10.3389/fphys.2021.667828 (PMC8264663; doi:10.3389/fphys.2021.667828)
Supplement: Supplementary file 1 [file Data_Sheet_1.PDF]

Supplementary Material

1 PROBABILITY DENSITY FUNCTION PER SLICE

In this section we show the PDF's per slice as described in the main text. We took 20 slices of size 0.05 in the direction $y = x$ for the plots shown in Fig. 9 of the main text. We then compute the mean residual and standard deviation per slice. For each slice, we have also plotted a Gaussian distribution (red curve) for guidance purposes which has mean and standard deviation set to the mean residual and standard deviation per slice, respectively.

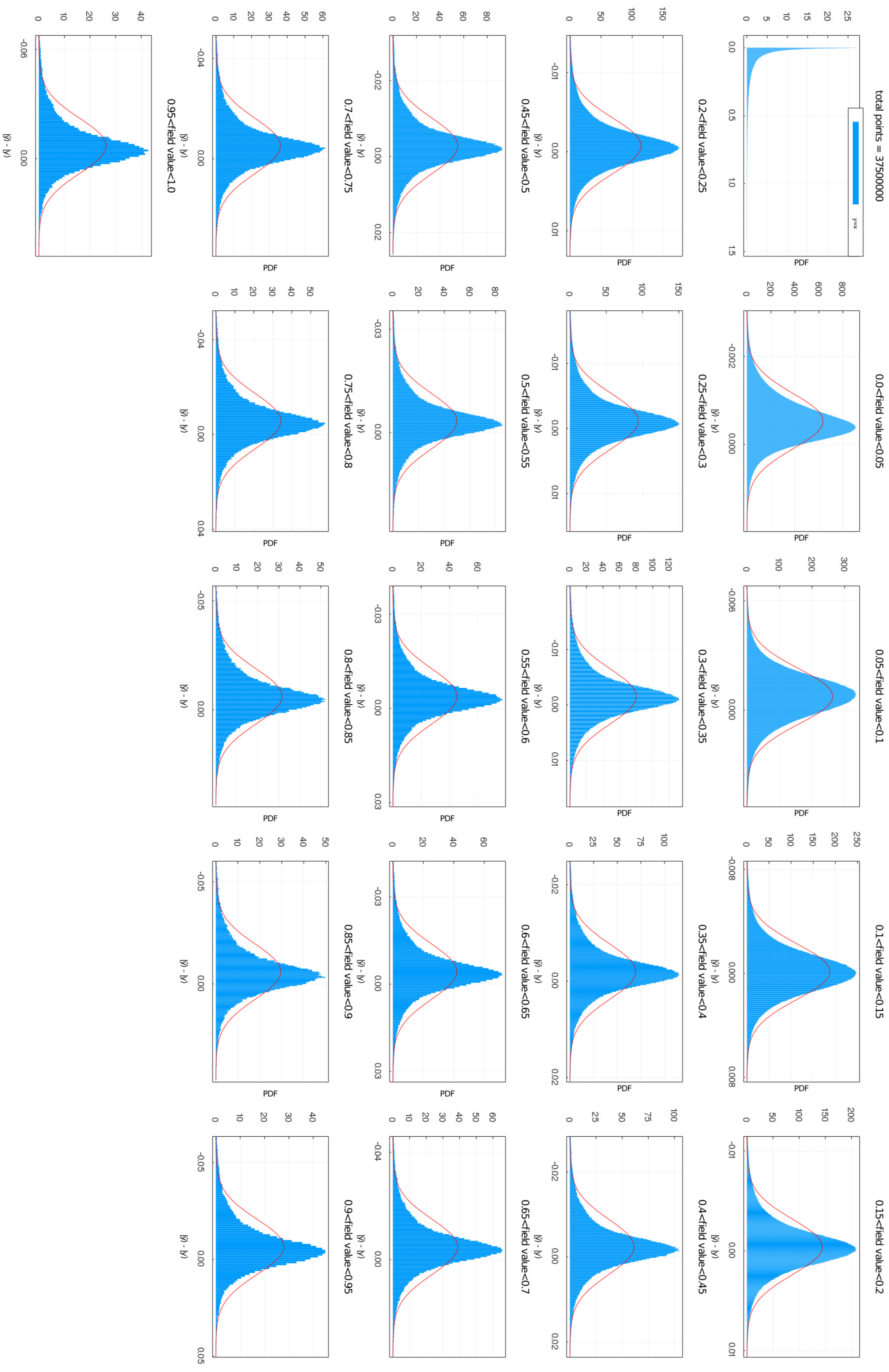

Figure S1. Model 1. PDF over all field values and PDF over slices. The red curve corresponds to a Gaussian distribution centered at the PDF mean value and with standard deviation equal to that of the PDF.

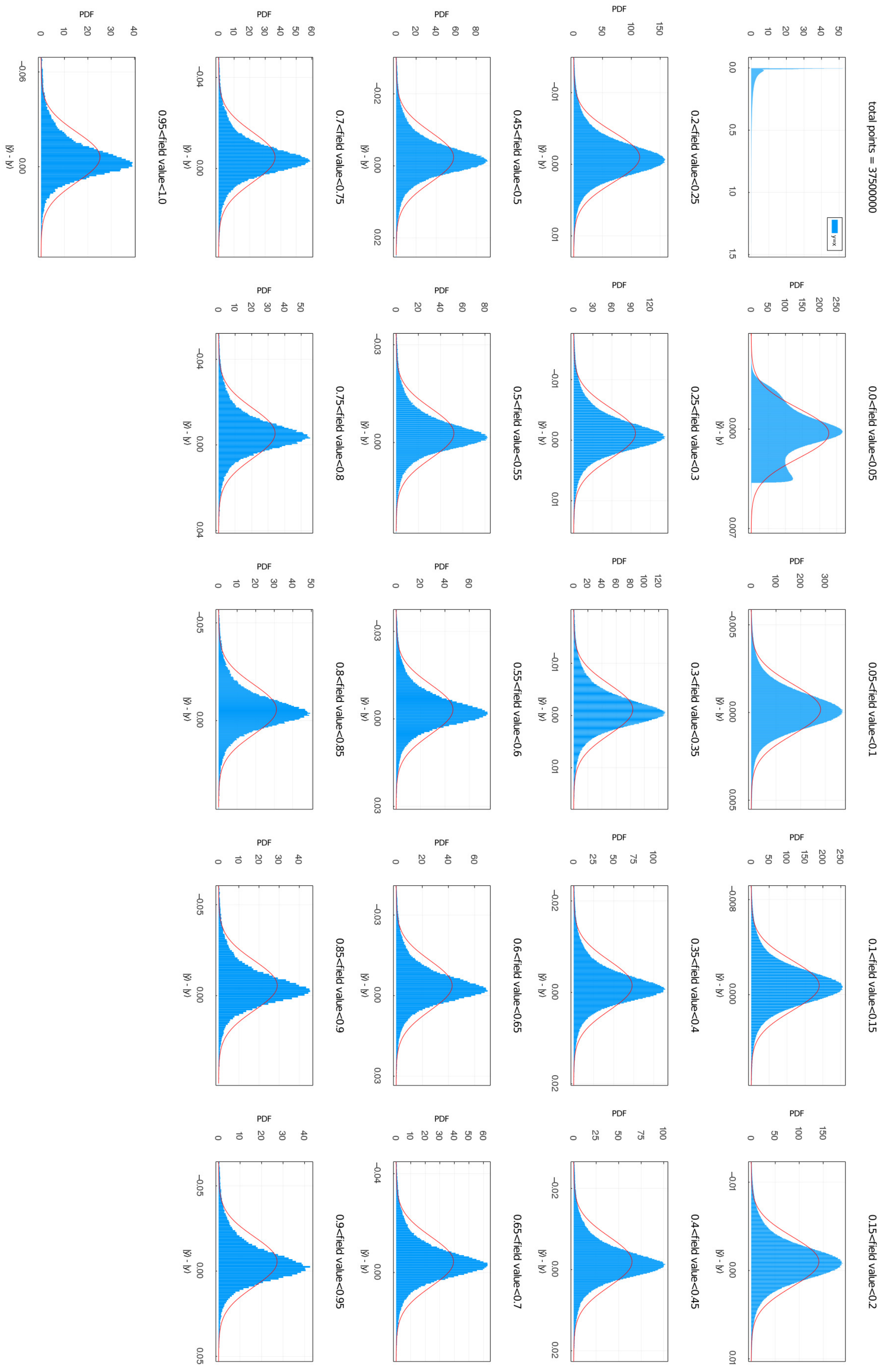

Figure S2. Model 2. PDF over all field values and PDF over slices. The red curve corresponds to a Gaussian distribution centered at the PDF mean value and with standard deviation equal to that of the PDF.

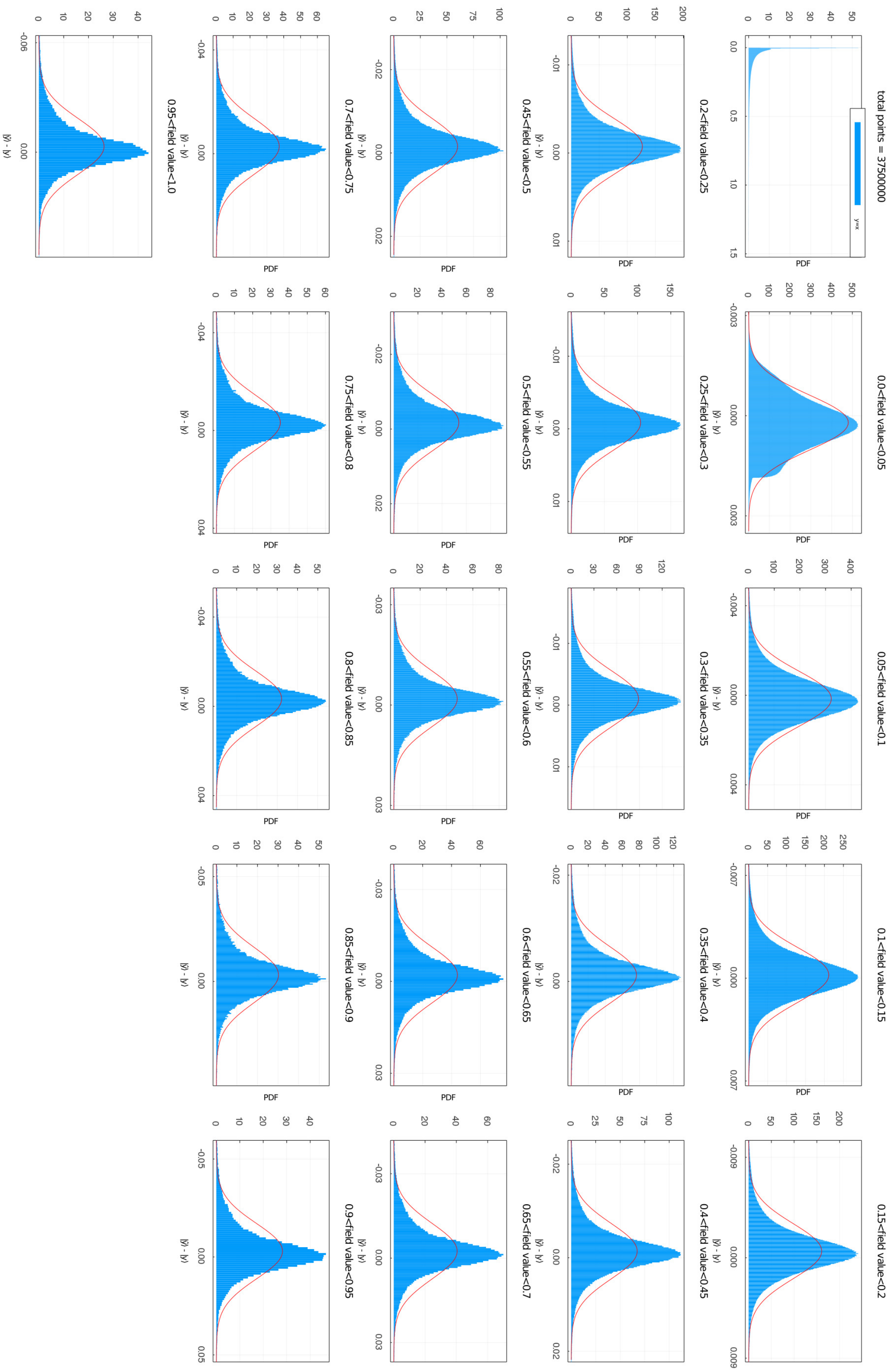

Figure S3. Model 3. PDF over all field values and PDF over slices. The red curve corresponds to a Gaussian distribution centered at the PDF mean value and with standard deviation equal to that of the PDF.

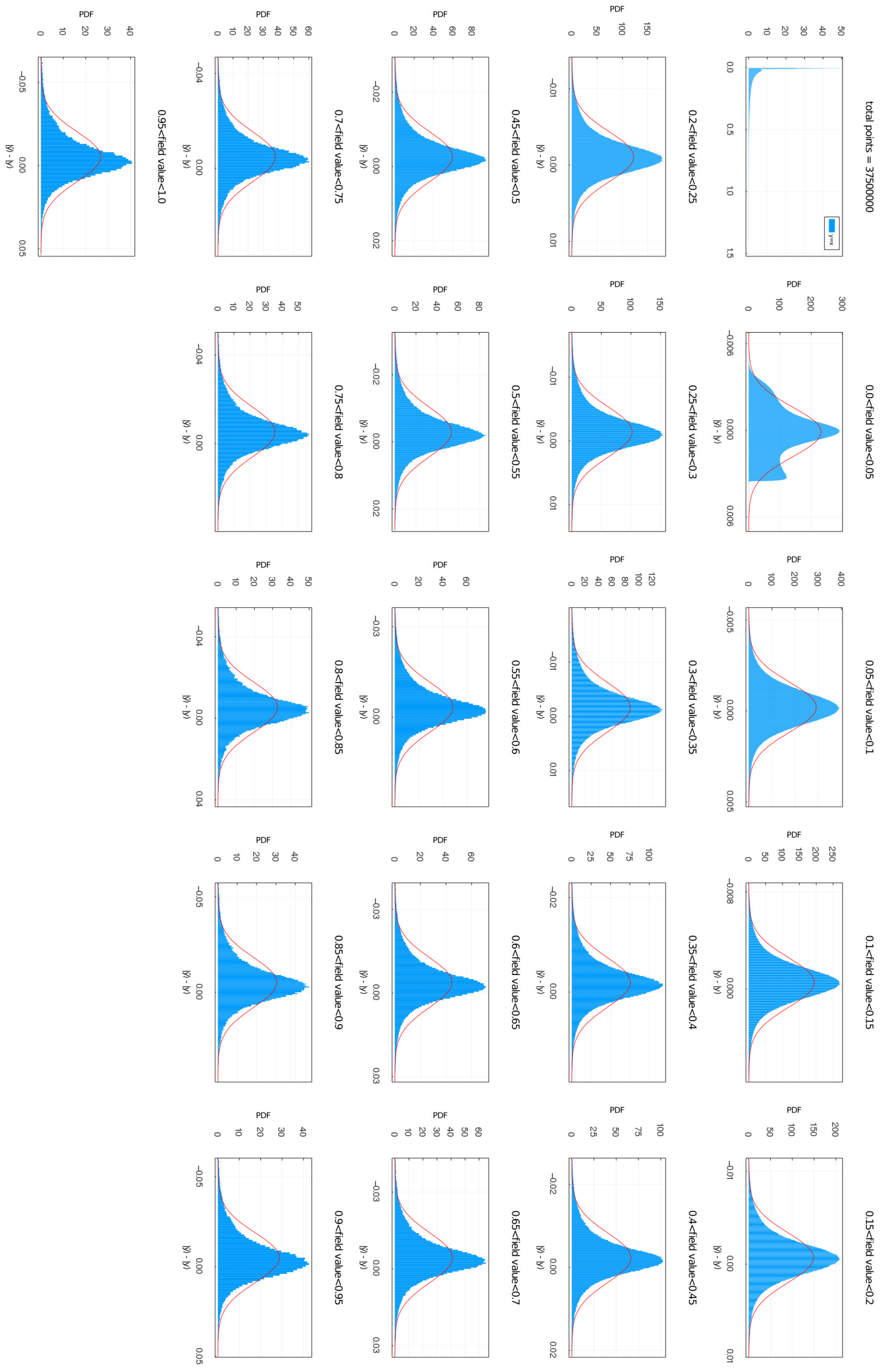

Figure S4. Model 5. PDF over all field values and PDF over slices. The red curve corresponds to a Gaussian distribution centered at the PDF mean value and with standard deviation equal to that of the PDF.

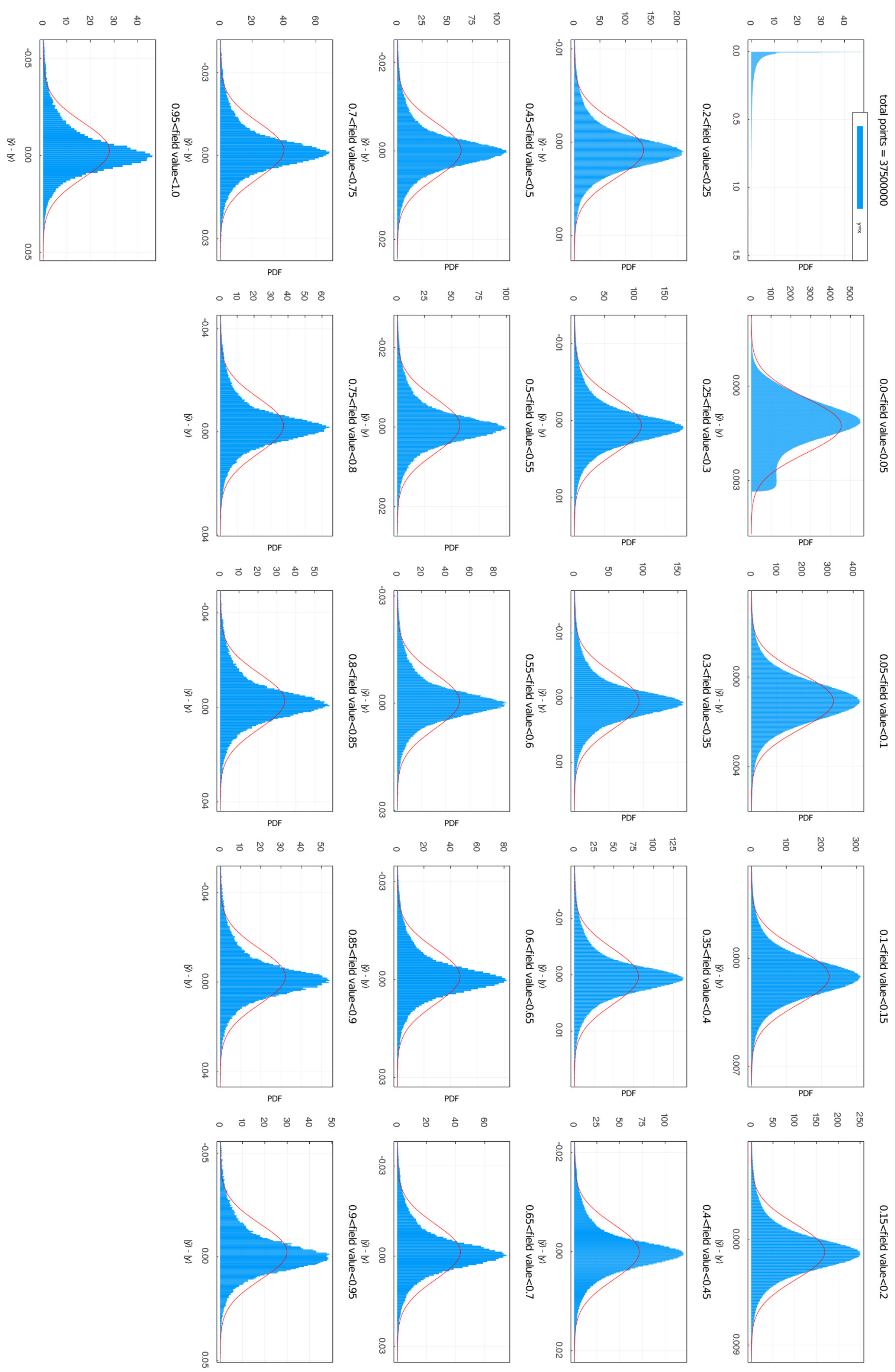

Figure S5. Model 6. PDF over all field values and PDF over slices. The red curve corresponds to a Gaussian distribution centered at the PDF mean value and with standard deviation equal to that of the PDF.

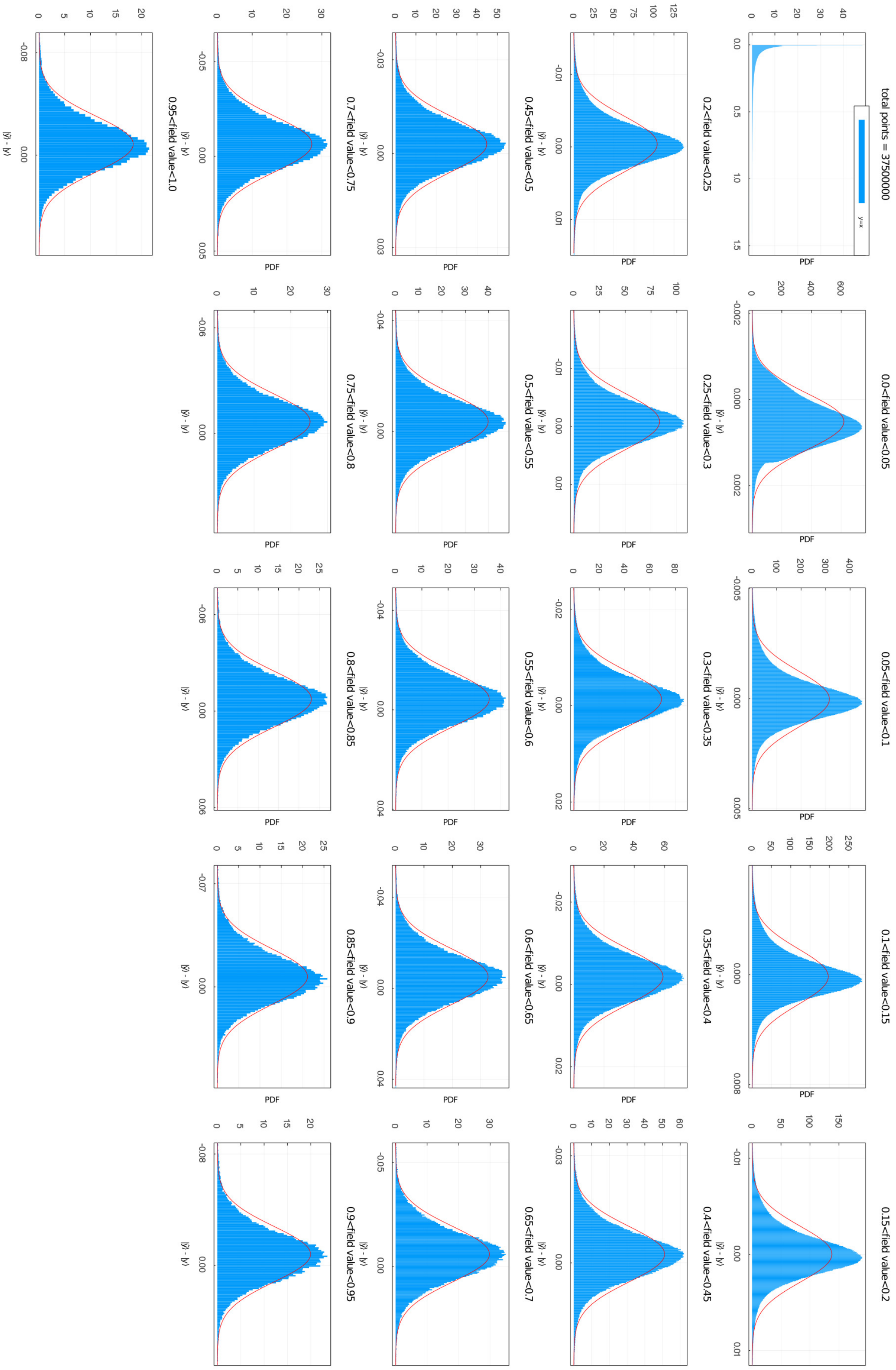

Figure S6. Model 8. PDF over all field values and PDF over slices. The red curve corresponds to a Gaussian distribution centered at the PDF mean value and with standard deviation equal to that of the PDF.

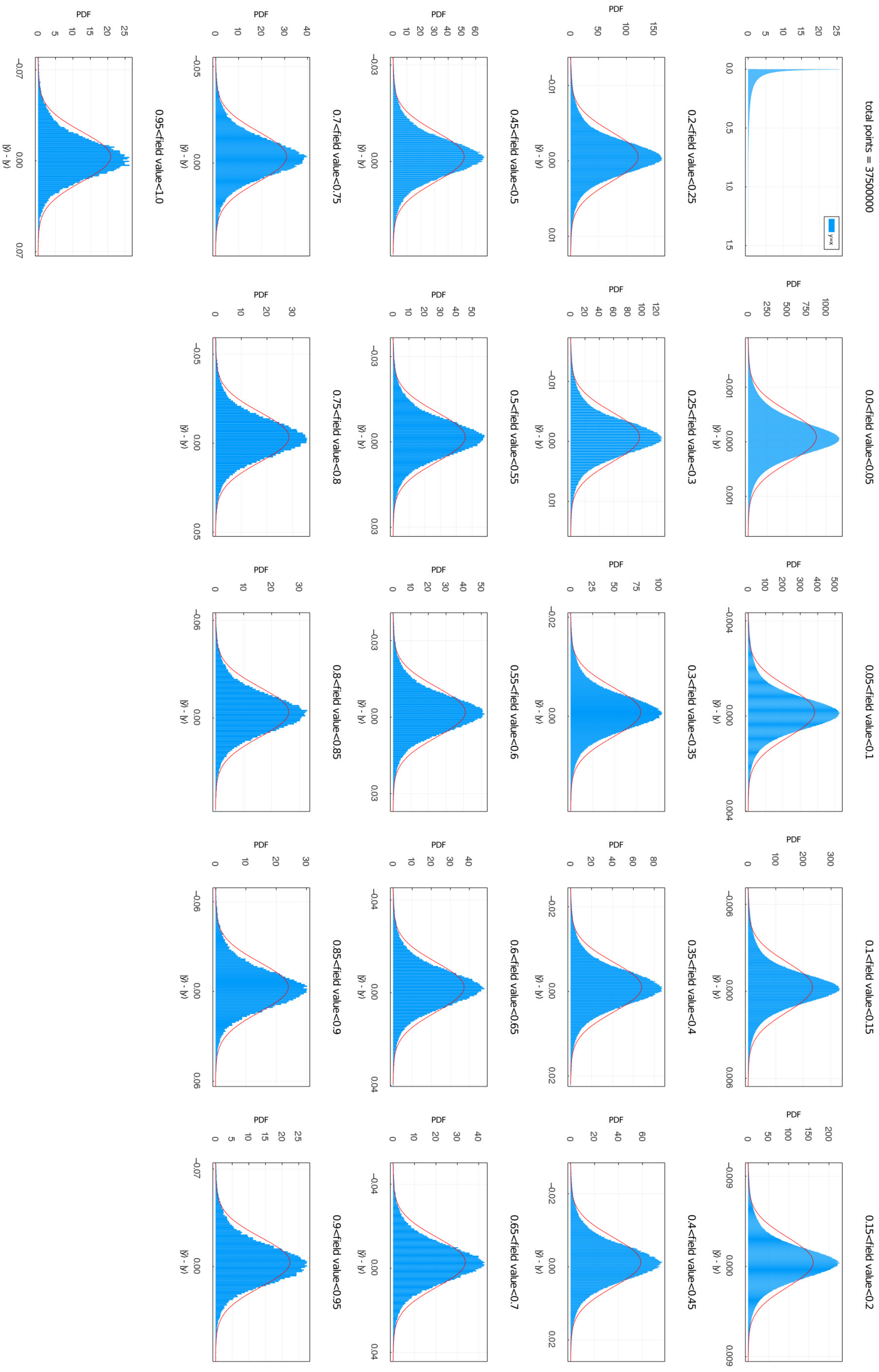

Figure S7. Model 9. PDF over all field values and PDF over slices. The red curve corresponds to a Gaussian distribution centered at the PDF mean value and with standard deviation equal to that of the PDF.

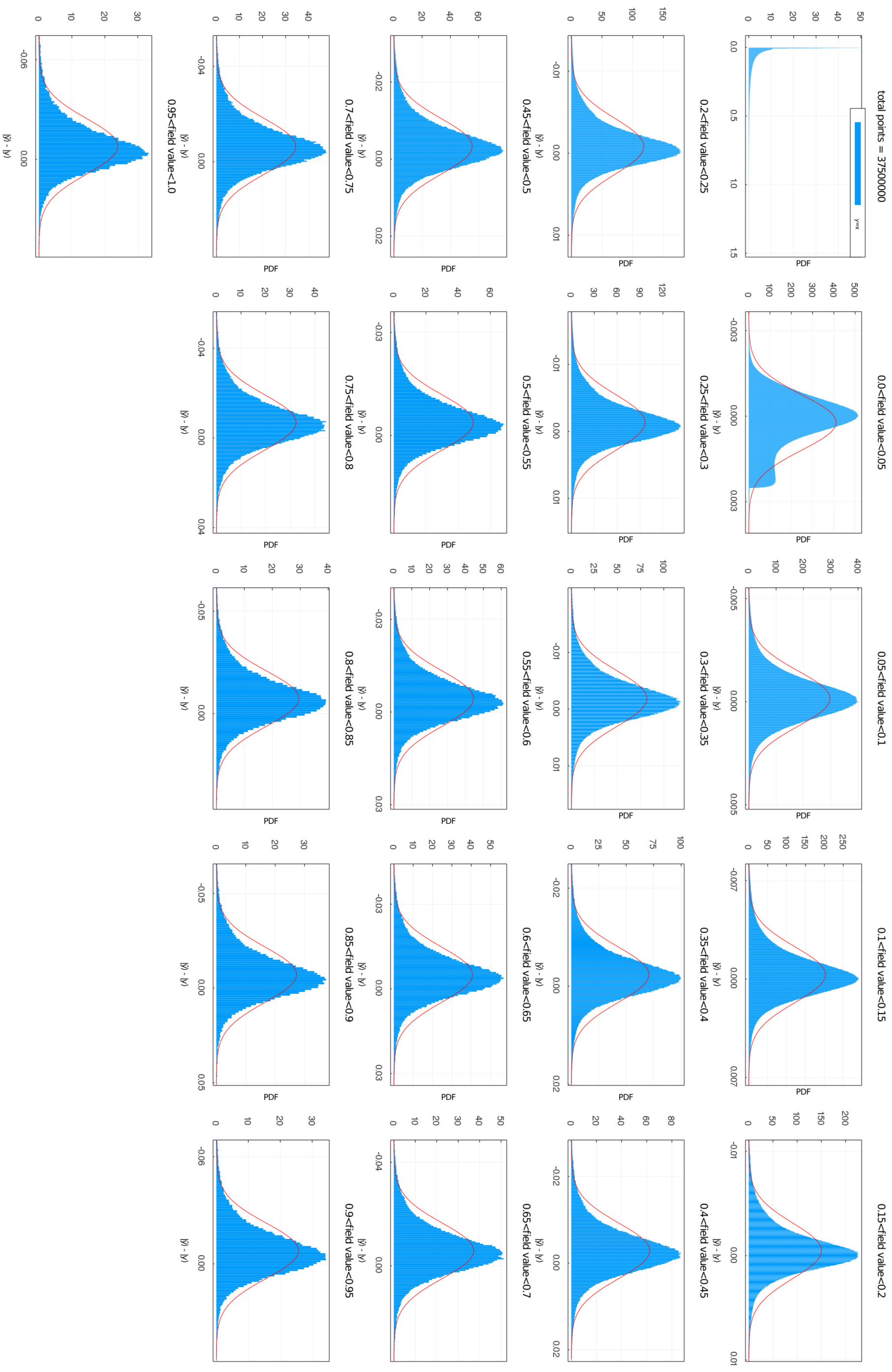

Figure S8. Model 10. PDF over all field values and PDF over slices. The red curve corresponds to a Gaussian distribution centered at the PDF mean value and with standard deviation equal to that of the PDF.

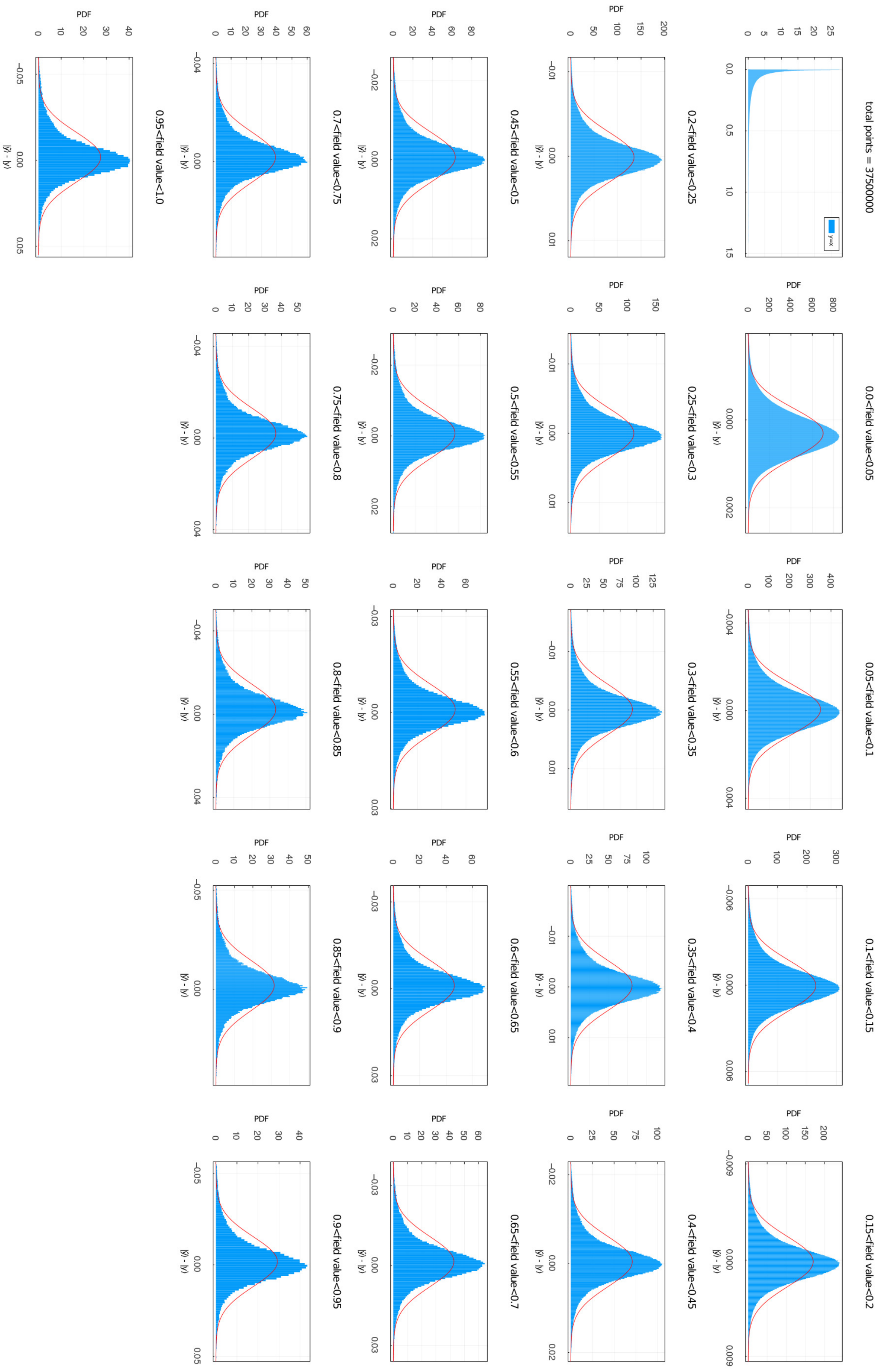

Figure S9. Model 11. PDF over all field values and PDF over slices. The red curve corresponds to a Gaussian distribution centered at the PDF mean value and with standard deviation equal to that of the PDF.

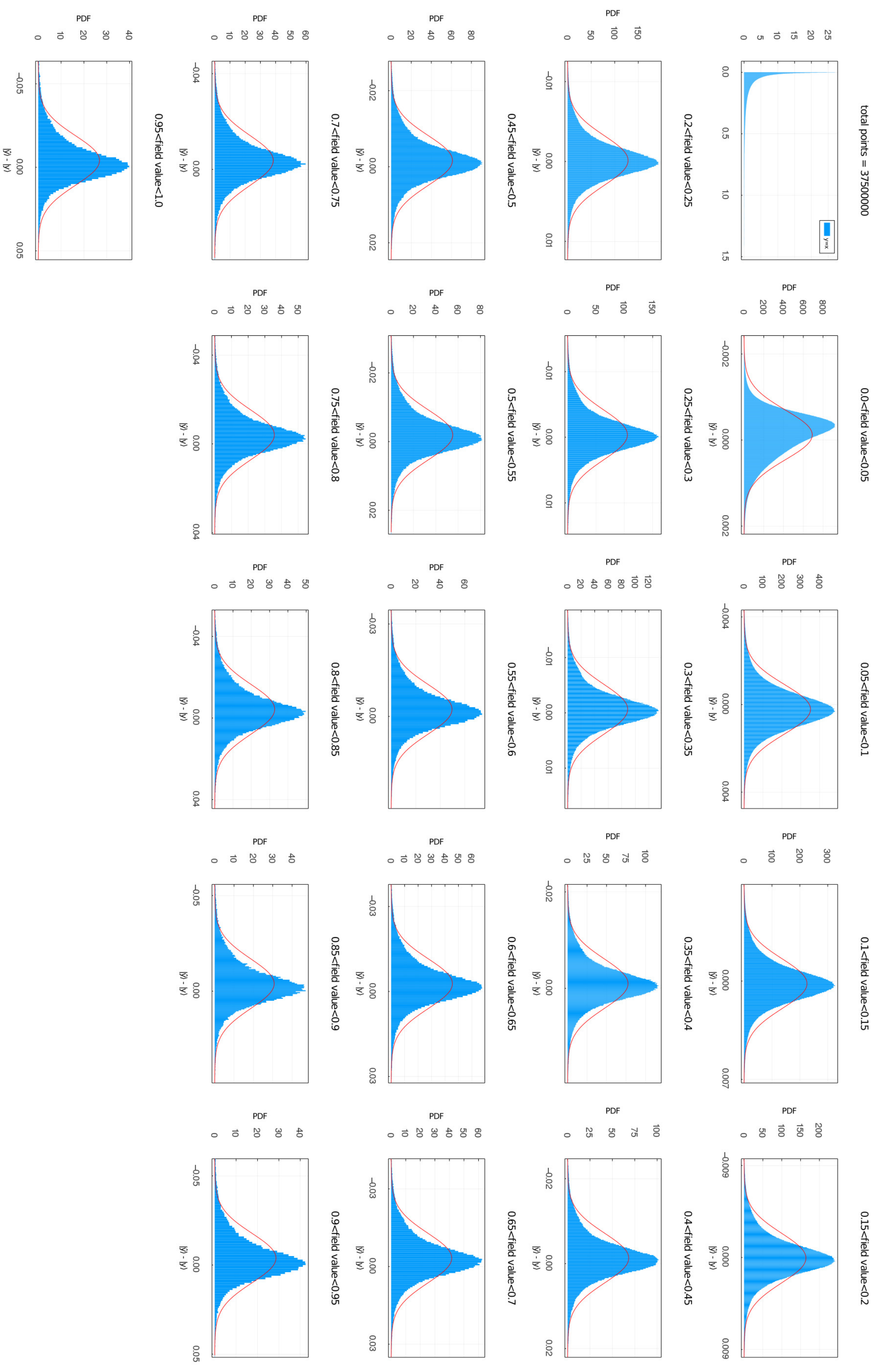

Figure S10. Model 12. PDF over all field values and PDF over slices. The red curve corresponds to a Gaussian distribution centered at the PDF mean value and with standard deviation equal to that of the PDF.

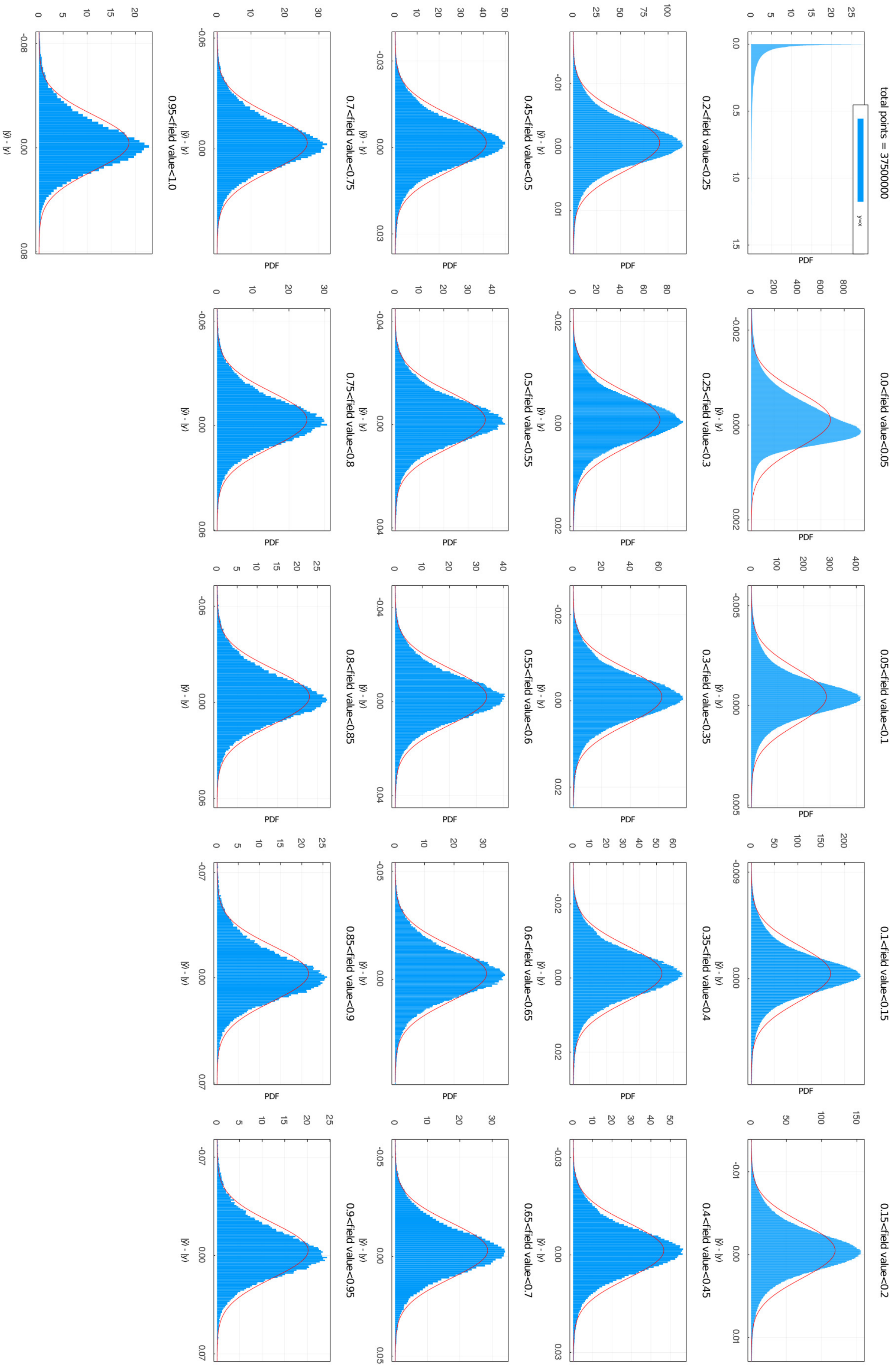

Figure S11. Model 13. PDF over all field values and PDF over slices. The red curve corresponds to a Gaussian distribution centered at the PDF mean value and with standard deviation equal to that of the PDF.

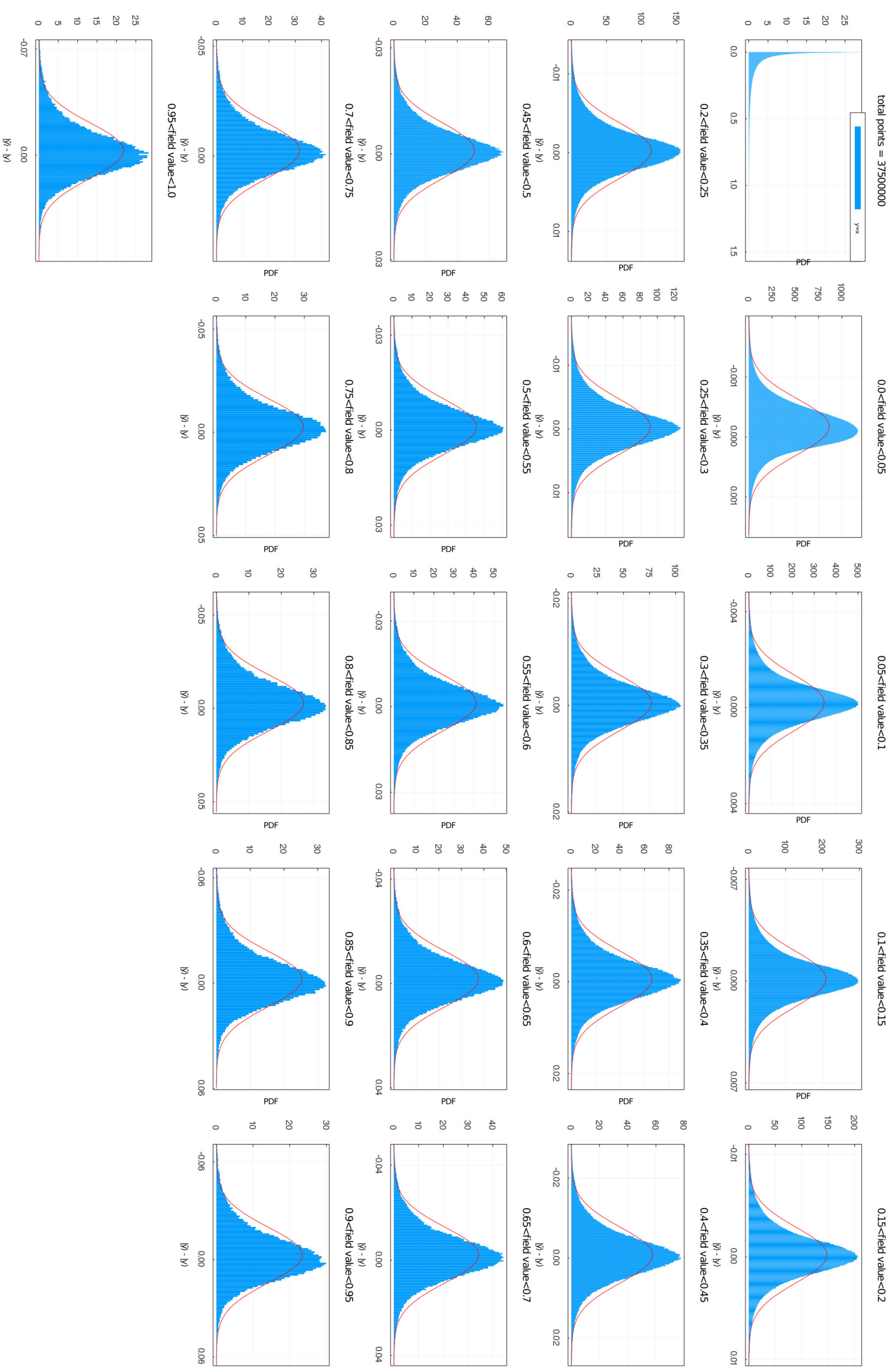

Figure S12. Model 14. PDF over all field values and PDF over slices. The red curve corresponds to a Gaussian distribution centered at the PDF mean value and with standard deviation equal to that of the PDF.
